# A stochastic model for dynamic reconfiguration of multi-microgrid networks under demand and supply uncertainties

**DOI:** 10.1038/s41598-026-52537-0

**Published:** 2026-05-19

**Authors:** Zakaria Yahia, Mohamed Gheith

**Affiliations:** 1 Department of Industrial and Manufacturing Engineering, Egypt-Japan University of Science and Technology, Alexandria, 21934 Egypt; 2 Department of Mechanical Engineering, Faculty of Engineering, Fayoum University, Fayoum, 63514 Egypt; 3 Production Engineering Department, Faculty of Engineering, Alexandria University, Alexandria, 21544 Egypt

**Keywords:** Microgrid, Reconfiguration, Load factor, Uncertainty, Sample average approximation, Energy science and technology, Engineering

## Abstract

Efficiently operating a single microgrid (MG) is increasingly challenging due to volatile electricity demand and intermittent renewable generation. Traditional static networks often fail to adapt to these fluctuations, compromising reliability. Incorporating these uncertainties into planning is essential for developing resilient optimization models that can withstand the stochastic nature of decentralized energy systems. This study proposes a dynamic reconfiguration strategy for interconnected microgrids that reroutes households based on real-time supply and demand. A stochastic nonlinear optimization model was developed to maximize load factors and flatten peaks while accounting for current-dependent power and distribution losses. The Sample Average Approximation (SAA) method was used to handle uncertainty, converting probabilistic variables into a robust deterministic equivalent that prioritizes electrical proximity during reconfiguration. The model was validated using a composite dataset spanning nearly two years of hourly load and renewable profiles. A total of 600 stochastic scenarios were considered and analyzed to represent an empirical distribution of real-world uncertainty while preserving key temporal correlations. Performance was tested under N-1 and N-2 contingency events, in which one or more microgrids are deactivated, to evaluate system resilience. Results indicate that while a single active MG improves the load factor, it also increases operational instability and objective function variance. Conversely, a three-MG configuration enhances system stability and predictability. Economically, the mesh architecture allows for temporary MG deactivation to reduce maintenance and fuel costs without compromising service. The proposed strategy achieves 100% resilience, ensuring uninterrupted service even under severe constraints.

## Introduction

Global electricity demand is growing rapidly, driven by economic growth, electrification, climate-related needs, and technological advancements. According to the International Energy Agency (IEA), electricity consumption is projected to expand at one of its fastest rates in decades during 2024–2025, with annual growth expected at approximately 4%, surpassing global GDP growth of 3.2% during the same period^1,2^.

The world’s electricity sector is growing significantly to meet rising demand. This growth is heavily focused on renewable energy, which accounted for over 90% of all new power added in 2024 – a record 585 GW, bringing the total to 4,448 GW^3^. Despite the rise of renewables, fossil fuels remain significant. From 2010 to 2023, coal-fired generation increased by nearly 2,000 TWh, and gas-fired generation rose by over 1,700 TWh^4^. To align with global climate goals to triple installed renewable energy capacity by 2030, renewable capacity must expand by 16.6% annually until 2030^3^.

To support the integration of expanding renewable energy resources, substantial investments are being directed toward modernizing power grids and deploying energy storage systems. These advancements are critical to maintaining supply reliability and mitigating the challenges posed by the intermittency of solar and wind generation. It is projected that over 1,700 GW of battery capacity will be added globally by 2035 to provide necessary dispatchable capacity (IRENA, 2025). Furthermore, long-term forecasts indicate that the world’s total electricity capacity may quadruple by 2050^5^.

The transition toward renewable energy integration poses significant technical challenges, primarily because solar and wind generation are intermittent. Consequently, intelligent grid management strategies and energy storage systems are required to maintain system stability. This large-scale shift also depends on stable policy frameworks and sustained investments to accelerate the move toward clean energy and achieve global generation targets. Historically, rising electricity demand was managed through a “chasing” strategy that relied on expanding centralized power stations to ensure supply adequacy. However, because approximately 20% of installed capacity is used only during peak demand periods—which occur for just 5% of the time—reliance solely on capacity expansion is no longer considered a viable or efficient strategy for meeting demand growth^6^.

To better balance electricity supply and demand, policymakers and utility companies are increasingly adopting several key strategies. Among these strategies is Demand Side Management (DSM), which includes mechanisms such as dynamic pricing, where electricity costs are adjusted based on the timing of consumption^7^. Furthermore, Demand Response (DR) programs provide incentives to reduce energy use during peak demand periods^8^. They’re also expanding clean energy sources like solar and wind power, incorporating smaller, local power sources (Distributed Energy Resources, DER), and upgrading power grids with smart technologies and improved ways to move electricity over long distances^9^.

This operational flexibility has been further advanced through the integration of Battery Storage Systems to mitigate renewable intermittency and capture the complex spatio-temporal co-variability inherent in renewable-dominant grids^10^. The potential of shared multi-energy storage has been explored in smart neighborhoods, where it functions as a coordinated cloud-based energy storage system to enhance participation across day-ahead, peer-to-peer, and hydrogen markets^11,12^. Simultaneously, market-driven strategies have evolved to empower microgrids as price-makers through strategic bidding in wholesale electricity markets and Peer-to-Peer trading frameworks^13^. Further sophistication is evident in the development of multi-carrier energy hubs, which integrate hydrogen and renewable sources using advanced uncertainty management strategies—such as Information Gap Decision Theory (IGDT)—to optimize residential energy performance and cost robustness^14^.

Micro Grid Reconfiguration (MGR) is proving to be a game-changer for load management. It enables flexible adjustments to how local power networks are connected^15^. Compared with earlier approaches, MGR allows consumers to maintain their daily routines without the disruption or inconvenience associated with load scheduling, while the utility can still provide stable electrification with a high load factor and without blackouts. Multi-Microgrid (MMG) Network Reconfiguration (NR) refers to the dynamic optimization of interconnection topologies and power flow paths among geographically proximate microgrids, enabling coordinated energy sharing, loss reduction, and resilience enhancement under uncertainty. Unlike a single MGR, which adjusts internal switches and DER dispatch, MMG Reconfiguration (MMGR) optimizes inter-microgrid ties, allowing surplus renewable generation in one microgrid to compensate for deficits in another while maintaining voltage stability and operational constraints. This capability is critical for modern distribution systems, where clusters of microgrids operate in grid-connected or islanded modes to mitigate renewable intermittency and demand volatility^16^.

**Fig. 1:**
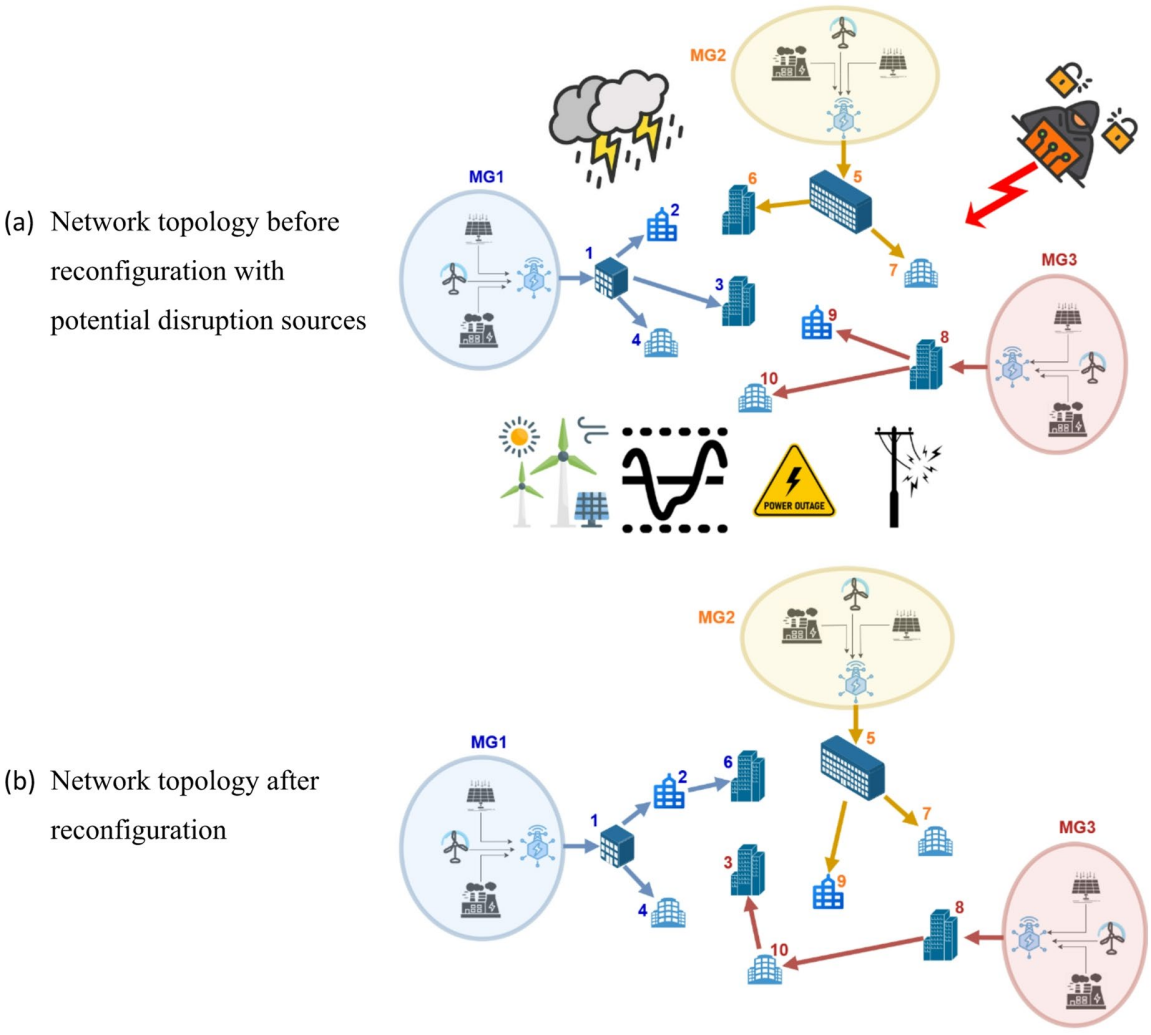
Network topology before and after reconfiguration with potential disruption sources.

The following schematic (Fig. 1) illustrates an example of the MMGR problem with MMGs and multiple households. Assume there are three microgrids, each with its own power DER, such as traditional fossil energy sources and/or renewable energy sources like wind turbines and/or solar panels. This network has ten households. The original status (Fig. 1a) shows that each household set is initially connected to a microgrid. Households 1–4 are supplied by MG 1, households 5–7 are supplied by MG 2, and the remaining households 8–10 are supplied by MG 3.

As illustrated, potential disruption sources could be external cyberattacks, natural crises, technical failures/faults, power outages, any variations in the renewable DER due to weather conditions (cloudy/radiation or windless/windy weather, the generation from solar panels and wind turbines falls), and/or changes in variations in households’ consumption load profile/pattern.

Under any of these disruption sources, and on an hourly basis throughout the day, the decision of which household is assigned or reassigned to one of the three microgrids and the associated route/link to be activated or deactivated is made. As an example of network reconfiguration, the topology after reconfiguration (Fig. 1b) shows that household 6 was initially connected to microgrid MG 2 and was reconnected to MG (1) Moreover, household 3 was initially connected to microgrid MG 1 and was reconnected to MG 3. Similarly, household 9 was initially connected to microgrid MG 3 and was reconnected to MG (2) This dynamic reconfiguration of each microgrid is intended to ensure that the combination of household loads fits the energy generation capacity of the associated microgrid.

By implementing a collaborative reconfiguration framework, existing household consumption patterns are preserved without interruption, and a stable power supply is maintained. This strategy enables operation at an optimal load factor while significantly enhancing system reliability by mitigating blackouts.

**Fig. 2:**
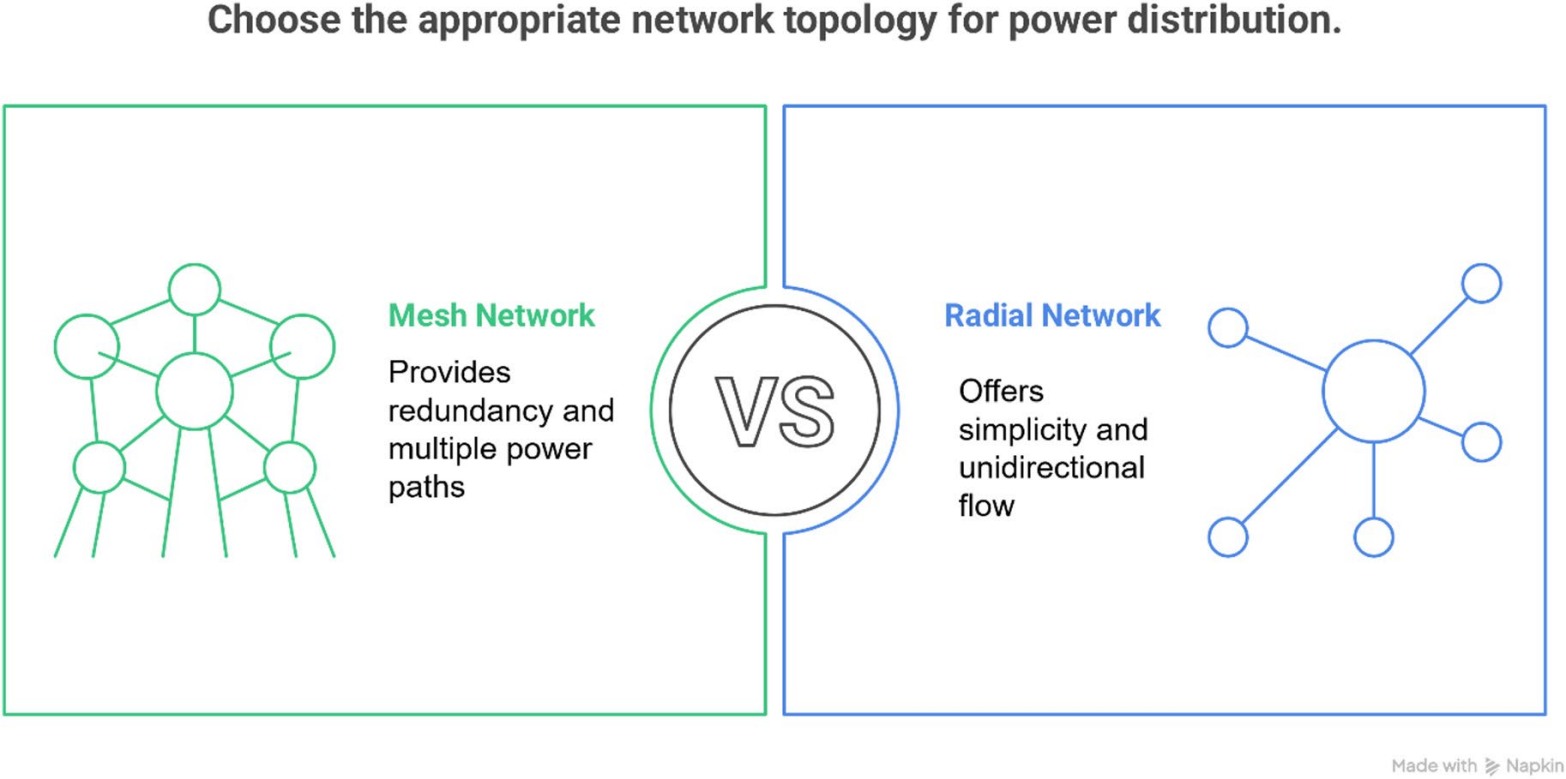
The structural differences between mesh and radial networks.

The structural differences between mesh and radial networks yield distinct reliability and efficiency trade-offs. Mesh networks, characterized by interconnected loops and multiple power-flow paths (Fig. 2), provide superior reliability through inherent fault tolerance, enabling automatic load redistribution during outages via tie-lines^17,18^. Their design also enhances efficiency by reducing power losses and improving voltage regulation through optimized load balancing^17^. In contrast, radial networks employ a simpler tree-like topology with unidirectional power flow (Fig. 2), making them cost-effective to implement but vulnerable to single-point failures^17^. While mesh configurations offer technical advantages, their sophisticated infrastructure requires greater capital investment and advanced protection systems. Radial networks remain preferable for cost-sensitive applications where high reliability is less critical.

The NR problem is addressed in mesh-connected MMG systems by using dynamic hourly binary decisions to assign households to specific microgrids. This optimization aims to maximize each microgrid’s load factor index while ensuring that all household hourly demands are met and microgrid generation capacities are respected. To maintain operational stability, the framework enforces a constraint that each household must be supplied by exactly one microgrid at any given time interval.

This research presents three primary technical contributions: First, a stochastic Mixed-Integer Nonlinear Programming (MINLP) optimization model is developed for mesh-connected microgrid networks to solve the dynamic, proactive reconfiguration problem under uncertainty. The model aims to maximize individual microgrid load factors and flatten peak demand while guaranteeing uninterrupted household consumption and accounting for distribution losses. The Sample Average Approximation (SAA) method is integrated into the framework to effectively manage stochastic variations in load and renewable generation.

Second, a comprehensive benchmark dataset is established for the Mesh Network Reconfiguration Problem. This dataset includes hourly stochastic demand and capacity scenarios for households and microgrids, enabling standardized comparisons for future research in the field.

Third, the performance of the proposed optimization model and system reliability are rigorously evaluated across various experimental scenarios. A detailed post-optimality analysis is conducted to assess the impact of microgrid shutdowns, providing a dual-perspective analysis of multi-microgrid performance from technical and financial standpoints.

**Fig. 3:**
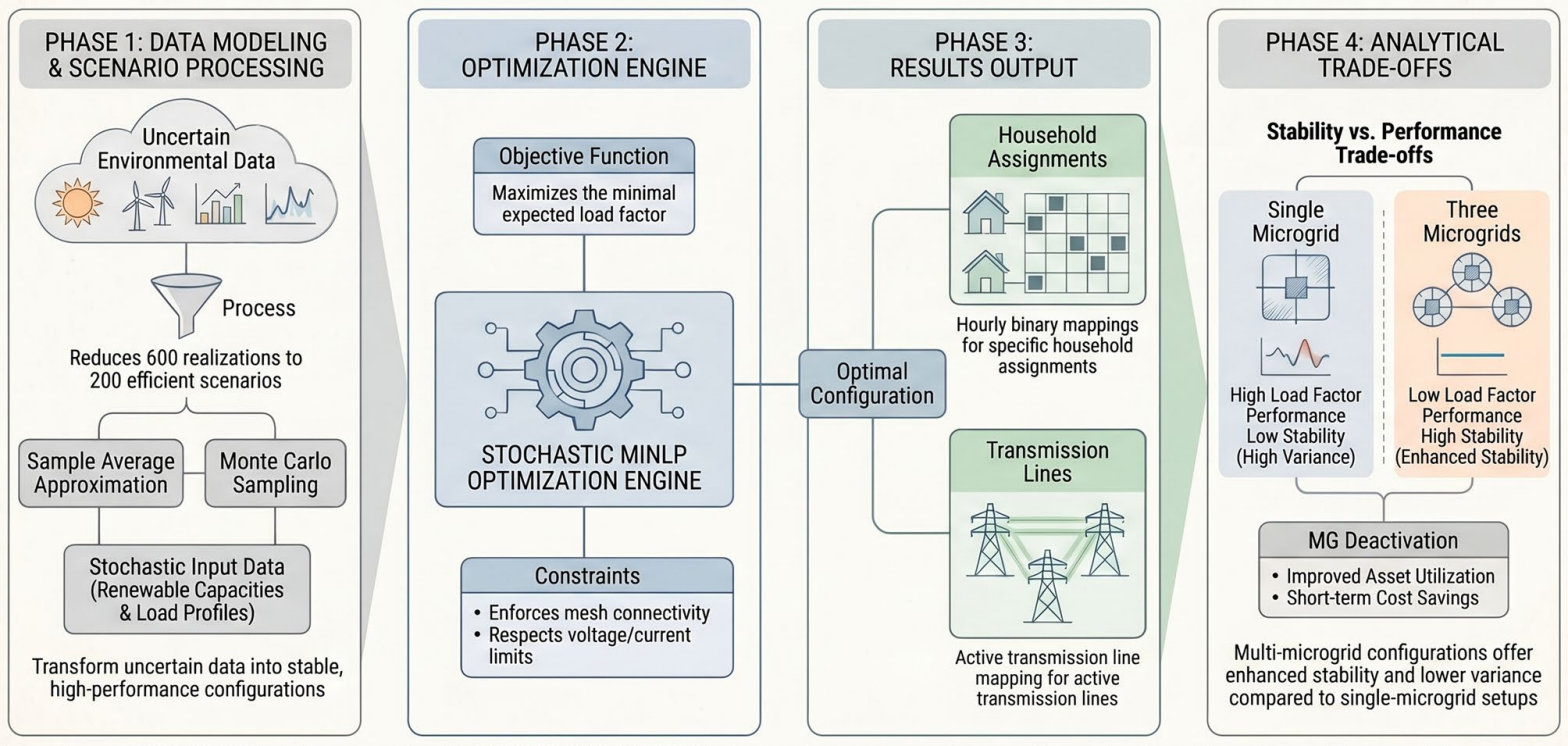
Schematic blueprint of the proposed stochastic multi-microgrid reconfiguration framework.

A schematic blueprint of the proposed stochastic MMGR framework is shown in Fig. 3. The framework is organized into four distinct phases: (1) Data Modeling and Scenario Processing, (2) Optimization Engine and Decision Output, (3) Optimal Configuration and Metrics, and (4) Configuration and Stability Trade-offs. While Phases 1 through 3 detail the stochastic processing and the optimization engine, Phase 4 provides a framework for a critical evaluation of the fundamental stability-efficiency trade-offs inherent in mesh-connected networks. This phase examines the operational ‘cons’ of different settings, such as the high operational variance that characterizes consolidated single-microgrid configurations. By incorporating this critical assessment into the research design, the study seeks to optimize a decentralized mesh architecture that functions as a ‘distributed buffer’ to mitigate demand-side disruptions. This visualization provides the structural roadmap for the transition from resource scheduling to the dynamic architectural reconfiguration examined in the subsequent sections.

The remainder of this paper is organized as follows: Sect.  2 summarizes the relevant literature review, highlighting research gaps and emphasizing the academic contribution of this study. Section  3 focuses on defining the problem and describes the proposed MINLP optimization model. Section  4 presents the case study and the complete data set used in this paper. Results, comparisons, and experimental analysis are presented and discussed in Sect.  5. Conclusions and future research directions are discussed in Sect.  6.

## Literature review

The NR in smart distribution systems has been extensively studied to optimize power flow, minimize losses, and enhance voltage stability. Recent reviews, such as the comprehensive work by El-Fergany^19^, consolidate advances in computational methods for NR, highlighting traditional, heuristic, meta-heuristic, and machine learning-based approaches. While these studies provide valuable insights into static and deterministic reconfiguration, they also identify critical gaps in addressing dynamic and stochastic environments—particularly in multi-microgrid systems with uncertain demand and renewable generation. This study bridges these gaps by proposing a stochastic model for dynamic multi-microgrid reconfiguration that explicitly incorporates demand and supply uncertainties.

Prior research is organized into three thematic clusters within this literature review: (1) deterministic reconfiguration studies, (2) dynamic reconfiguration methods addressing hourly load and generation profiles, and (3) stochastic reconfiguration methods addressing either temporal variations or uncertainty. A comprehensive summary of the related literature is provided in Table 1. Based on this classification, critical gaps in existing methodologies are identified, specifically the absence of integrated stochastic-dynamic frameworks for mesh network reconfiguration. This study subsequently presents novel contributions designed to address these research shortcomings through a unified optimization framework.

Nguyen et al.,^20^ proposed an adaptive cuckoo search algorithm integrated with graph theory to address the Distribution Network Reconfiguration (DNR) problem and optimize Distributed Generator (DG) placement and sizing, aiming to minimize active power losses and improve voltage stability in radial distribution systems. Similarly, Muthukumara & Jayalalitha^21^ proposed a deterministic optimization framework for radial DNR that combines NR, DG placement, and capacitor sizing to minimize active power losses and improve voltage profiles. The approach uses a hybrid Harmony Search Algorithm and Particle Artificial Bee Colony (HSA-PABC) algorithm, leveraging HSA’s exploration and PABC’s exploitation to achieve faster convergence and superior solution quality compared to standalone methods. Hemmatpour et al.,^22^ proposed a deterministic multi-objective optimization framework for Islanded microgrids (IMGs), introducing a novel voltage stability index. They developed an Adaptive Multi-Objective Harmony Search Algorithm (AMOHSA) that simultaneously minimizes active power losses and maximizes voltage stability.

Camacho-Gómez et al.,^23^ presented a deterministic multi-objective optimization framework for jointly designing microgrid topologies and locating renewable resources (PVs and micro-wind turbines) in small-scale systems (8–12 nodes). The study minimizes network deployment costs and electrical power losses simultaneously using an HSA. Agrawal et al.,^24^ presented a three-stage self-healing algorithm designed to improve the resilience of active distribution systems during severe faults, such as multiple line outages or blackouts. The approach avoids intentional islanding and instead focuses on NR to maximize critical load restoration. The method first identifies feasible loads to energize, then optimizes the network topology using Genetic Algorithms (GA) for efficient reconfiguration. Similarly, Shi et al.,^25^ presented a deterministic outage management strategy that combines NR and DER scheduling to enhance distribution system resilience during extreme weather events. The approach first identifies feasible radial topologies through incidence matrix ranking, then solves linear programming problems to optimally schedule dispatchable (microturbines) and non-dispatchable (PV) DERs while minimizing operational costs and load-shedding penalties.

Fathi et al.,^26^ proposed a deterministic multi-criteria optimization approach for simultaneously allocating PVs and wind turbines and reconfiguring a radial distribution network, aiming to minimize active power losses and reliability improvement costs. They developed an Improved Slap Swarm Algorithm (ISSA) enhanced with Differential Evolution operators to optimize renewable resource locations, sizes, power factors, and tie-line switching states of renewable resources. Similarly, Rahiminejad et al.,^27^ developed a deterministic, multi-objective, scenario-based resilience enhancement framework for IMGs against cyberattacks. The Teaching-Learning-Based Optimization (TLBO) algorithm is applied to maximize resilience and minimize worst-case load curtailment while optimizing both long-term infrastructure design (to reduce attack likelihood/impact) and dynamic network reconfiguration (to maintain critical load supply during and after attacks).

Hosseini et al.^28^ developed a deterministic multi-objective optimization model for microgrid-integrated distribution systems, combining techno-economic energy scheduling with network reconfiguration and flexible energy trading to simultaneously minimize power losses, maximize trading profits, and enhance reliability. The resulting MINLP was solved using Branch and Bound with fuzzy satisfaction-based trade-off analysis. Scenario-based reserve planning was employed to handle renewable generation and load uncertainties while optimizing hourly energy transactions in a modified IEEE 33-bus test system. Long et al.^29^ presented a deterministic optimization approach using Evolutionary Particle Swarm Optimization (EPSO) to enhance distribution network efficiency through reconfiguration, targeting power loss minimization under varying load conditions. The EPSO algorithm, which combines Evolutionary Programming and PSO, outperforms traditional methods in convergence speed and solution quality while enforcing radiality constraints, voltage limits, and load variability through load flow analysis.

Few studies have considered dynamic Distribution Network Reconfiguration on an hourly basis. Esmaeili et al.^30^ developed a deterministic optimization framework for smart distribution systems, combining day-ahead scheduling with hourly network reconfiguration to minimize total operational costs. Using Particle Swarm Optimization (PSO) to solve the MINLP problem, the approach optimizes system performance based on forecasted load profiles, market prices, and generation capacities while accounting for protection device constraints. Jafari et al.^31^ introduced a hybrid metaheuristic algorithm (combining Wild Goats Algorithm and Exchange Market Algorithm) for deterministic dynamic reconfiguration of distribution networks, targeting power loss reduction and reliability enhancement. Ismail et al.^15^ proposed a deterministic Mixed-integer Linear Programming (MILP) framework for reconfiguring multi-microgrid systems to maximize load factors, optimizing power utilization efficiency while accounting for generation capacities, household demands, and distribution losses. Most recently, Nguyen and Nguyen^32^ presented a deterministic hybrid optimization framework for dynamic distribution network reconfiguration, targeting power loss minimization, voltage stability improvement, and load balancing in systems with high penetration of renewables (PV/wind) and electric vehicles. The study developed a hybrid algorithm, combining Particle Swarm Optimization’s local search efficiency with Grey Wolf Optimizer’s global exploration capabilities to solve the nonlinear multi-objective problem. Using weighted random allocation of normalized objectives (losses, voltage deviations, load variance), the approach demonstrates superior convergence and solution quality compared to standalone methods.

Only a limited number of studies have investigated stochastic Distribution Network Reconfiguration under uncertainties in load demand and energy resource generation. Esmaeili et al.^33^ proposed a stochastic multi-objective optimization framework for DNR and DG allocation, incorporating load uncertainty via Triangular Fuzzy Numbers to reflect real-world variability. The study simultaneously minimizes power losses, costs, and emissions while maximizing voltage stability, using a novel Multi-Objective Hybrid Big Bang-Big Crunch (MOHBB-BC) algorithm to explore Pareto-optimal solutions. Kianmehr et al.^34^ developed a multi-objective stochastic optimization framework for joint DG allocation and distribution network reconfiguration, balancing DG owner profits and operational costs under uncertainties in wind power, load demand, and electricity prices. Using the ε-constraint method and the Self-adaptive Bacterial Foraging (SBF) algorithm, the approach generates Pareto-optimal solutions, with a fuzzy satisfaction technique selecting the best compromise.

Uniyal and Sarangia^35^ proposed a stochastic optimization framework for reconfiguring multi-microgrid networks, explicitly addressing uncertainties in renewable generation and load demand. They minimized voltage variations and power losses while incorporating probabilistic representations of system variables through scenario-based stochastic programming and point estimate methods. The solution leveraged an adaptive modified whale optimization algorithm to handle combinatorial complexity and integrated probabilistic load flow analysis for robustness. Najafi and Miveh^36^ proposed a multi-objective stochastic optimization framework for simultaneous PV placement and distribution network reconfiguration, addressing three objectives: (1) maximizing PV owner profits, (2) minimizing DisCo operational/reconfiguration costs, and (3) reducing expected interruption costs (ECOST). The model incorporated uncertainties in PV generation, electricity prices, and load demand via scenario-based stochastic programming. Using the Dragonfly Algorithm (DA), the study generated Pareto-optimal solutions, with a fuzzy satisfaction method selecting balanced compromises.

Existing research on microgrid reconfiguration exhibits several critical limitations. First, while deterministic models offer computational efficiency, they neglect the inherent uncertainties in demand and renewable generation, leading to reactive rather than proactive network management. Most stochastic studies focus narrowly on cost/loss minimization or DG profitability. They often overlook integrated multi-microgrid dynamic load balancing, particularly through load factor optimization, and dynamic resilience in mesh-topology systems. Additionally, current work predominantly assumes radial networks, failing to address the decentralization potential of mesh configurations. These mesh configurations are vital for crisis-responsive reconfiguration, especially for stochastic, demand-side-integrated reconfiguration, where their redundancy and flexibility offer transformative potential. This transition is especially crucial as DERs require networks capable of bidirectional power flows and dynamic fault response—capabilities inherently embedded in mesh architectures but structurally unattainable in radial systems.

These gaps highlight a need for frameworks that combine stochastic and multi-period dynamic optimization, load-leveling objectives, and mesh-network adaptability under real-world variability.

A stochastic MINLP model is developed to address identified research gaps in the dynamic reconfiguration of mesh-connected MMG systems. This framework integrates the SAA method to simultaneously optimize load factors and operational constraints under uncertainty. Key technical innovations of this study include:

1. Proactive uncertainty management is achieved through probabilistic modeling of stochastic demand and fluctuations in renewable generation.
2. Peak load leveling is achieved through linearized load-factor optimization while strictly maintaining distribution-loss constraints.
3. A household-level mesh-topology reconfiguration strategy is implemented to provide superior decentralization and crisis resilience.

This architectural approach represents a fundamental departure from traditional radial networks, which are often vulnerable to single-point failures.

Dynamic hourly reconfiguration is enabled to accommodate stochastic supply-demand fluctuations, and a benchmarking framework utilizing a harmonized synthetic dataset is established to facilitate standardized performance evaluation. By adopting a mesh-topology MMG system—characterized by the integration of DERs, interconnected substations, and multi-directional power flow—system reliability and power quality are significantly improved during outages.

Unlike traditional radial networks, which are vulnerable to single-point failures, the mesh architecture provides superior reliability through inherent fault tolerance, enabling automatic load redistribution via tie-lines. This framework ensures that even when a primary supply path is compromised, service continuity is maintained through alternative routing options. The effectiveness of this approach in enhancing system resilience is further validated through the implementation of a stochastic optimization model that accounts for the inherent uncertainties in both household demand and renewable generation.

Together, these advances deliver a computationally efficient, uncertainty-aware solution for resilient microgrid operations, surpassing the limitations of static or radial-network approaches in existing literature.

**Table 1:** Related literature review summary.

References	Problem	Objective function	Approach	Hourly reconfiguration	Uncertainty consideration	Numerical case study data
Nguyen et al.^20^	DNR and Distributed Generation Units Allocation (DGUA).	Minimize the total power loss and voltage stability deviation index.	Cuckoo Search Algorithm.	×	×	33, 69 and 119 buses IEEE Test Systems.
Hemmatpour et al.^22^	IMGs Configuration.	Maximizing voltage stability and minimizing active power losses.	AMOHSA	×	×	33 and 69 buses IEEE Test Systems.
Muthukumara and Jayalalitha^21^	Network Topology Reconfiguration, DGUA.	Minimize power losses.	HSA and PABC	×	×	69 and 118 buses IEEE Test Systems.
Camacho-Gómez et al.^23^	Topology of MG network and optimally locating distributed renewable energy resources.	Minimize the network deployment cost and the electrical power losses.	HSA	×	×	Two small-sized MGs with PVs and Micro WTs: 8 nodes and 12 nodes.
Agrawal et al.^24^	Self-healing via NR.	Maximize priority loads restoration and Minimize feeder power loss.	Simulation and GA	×	×	33-buses IEEE Test System with Seven cases of faulty lines.
Shi^25^	NR and DER scheduling.	Minimize the total outage cost (the accumulative cost for dispatchable DER operation and load reduction).	Linear Programming and Heuristic Rules.	×	×	69 and 123 buses IEEE Test Systems.
Fathi et al.^26^	Allocation and Sizing of Renewable Energy Resources and NR.	Minimize the active power losses and the reliability improvement costs.	ISSA	×	×	33 and 69 buses IEEE Test Systems.
Rahiminejad^27^	DNR and DER Allocation and Sizing.	Minimize the maximum load curtailment across all attack scenarios.	TLBO Algorithm	×	×	17 and 69 buses IEEE Test Systems.
Hosseini et al.^28^	MGR and Energy Sell/Buy Trading.	Minimizing power losses and operational costs and maximizing energy trading profits.	Multi-objective MINLP, Branch and Bound	× (Trading only has been conducted on an hourly basis.)	× (Uncertainty analysis has been considered using reserve planning.)	33-bus IEEE Test System with four MGs.
Long et al.^29^	Distribution networks reconfiguration.	Minimize total power losses.	EPSO algorithm	×	×	33-bus IEEE Test System.
Esmaeili et al.^30^	Day-ahead scheduling and hourly distribution networks reconfiguration.	Minimize total cost.	PSO algorithm	✓	×	33-bus IEEE Test System.
Jafari et al.^31^	Dynamic and multi-objective reconfiguration of the distribution networks.	Minimize unreliability and power loss.	Hybrid Exchange Market Algorithm and Wild Goats Algorithm.	✓	×	15, 33, 69 and 85 buses IEEE Test Systems.
Ismail et al.^15^	Dynamic reconfiguration of multi-microgrid systems.	Maximize the load factor of each microgrid.	Deterministic MILP model.	✓	×	An artificial system with three microgrids, 15 households and PV, WT and diesel distributed units.
Nguyen and Nguyen^32^	Reconfiguration of distribution networks.	Minimizing power losses, voltage deviations, and load variance.	Hybrid PSO and Grey Wolf Optimization	✓	×	33-bus IEEE Test System.
Esmaeili et al.^33^	DNR and DG power allocation.	Minimizing power losses, maximizing voltage stability, reducing costs, and lowering emissions.	MOHBB-BC Algorithm	×	✓ (load demand uncertainty)	25 and 33 buses IEEE Test Systems.
Kianmehr et al.^34^	multi-objective joint reconfiguration of distribution networks and allocation of DG units.	Maximizing profit and minimizing cost.	Stochastic MINLP, ε-constraint method and SBF algorithm.	×	✓	33-bus IEEE Test System.
Uniyal and Sarangia^35^	Network Reconfiguration.	Minimize voltage variations and power losses.	Adaptive Modified Whale Optimization Algorithm.	×	✓	33 and 69 buses IEEE Test Systems.
Najafi and Miveh^36^	Placement of PV systems and reconfiguring DNs	Maximize PV owners’ profits, minimize the distribution company’s costs, and reduce ECOST	Multi-objective stochastic programming and DA	×	✓	33-bus IEEE Test System
This Study	Dynamic Stochastic reconfiguration of multi-microgrid systems	Maximize the load factor of each microgrid.	Stochastic MINLP model using SAA	✓	✓	An artificial data set with three microgrids and 15 households. Each microgridwith three power units: PV, WT and diesel unit

## The proposed mathematical model

A stochastic MINLP mathematical model is presented in this section to address inherent uncertainties in both household electricity consumption and renewable energy generation. This framework extends the deterministic linearized model previously developed by Ismail et al.^15^ to incorporate the new stochastic variants addressed in this study. The model is designed to solve the dynamic reconfiguration scheduling problem for MMG networks under uncertainty. The primary objective of the proposed model is to maximize the minimum expected load factor index across the multi-microgrid network to facilitate peak load leveling while ensuring that household consumption patterns remain undisturbed and that distribution losses are strictly respected. In the following subsections, the problem description will first be presented, accompanied by model assumptions, followed by the stochastic MINLP formulation using the SAA method, with a detailed description and graphical illustrations of the system constraints.

**Fig. 4:**
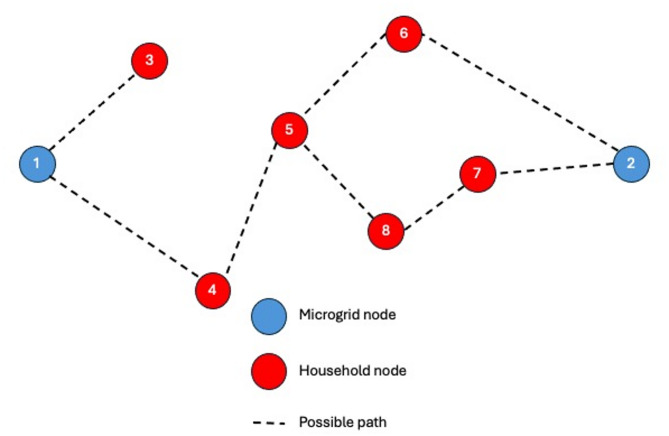
Network topology showing connections between household nodes and two microgrids for model visualization.

The proposed stochastic model addresses the reconfiguration problem under uncertainty in both household electricity consumption and the outcomes of renewable energy sources. The problem is formulated as an MINLP model using SAA. The problem is formulated for a network with a given number *N* of nodes. Where nodes include *M* microgrids or the supply nodes and *H* households or the demand nodes as shown in Fig. 4. The model is concerned with the main decision of assigning household (*h*) to a microgrid (*m*) at a time (*t*). The main decision variable decides whether a household *h* (or node $\:i$) is associated with microgrid *m* (or node *j*), which means that the power line or branch ($\:i,\:j$) is activated. Thus, the main decision variable is binary and is referred to $\:{x}_{ijmt}$, where it will be equal to one if the node $\:\left(i\right)$ is connected to node $\:\left(j\right)$ supplied from microgrid $\:\left(m\right)$ at time period $\:\left(t\right)$, and zero otherwise. To track the power source to a certain microgrid $\:\left(m\right)$, another binary decision variable $\:{y}_{imt}$ is considered, where it is equal to 1 if node $\:\left(i\right)$ is supplied from microgrid $\:\left(m\right)$ at time period $\:\left(t\right)$, and zero otherwise.

Figure 4 illustrates the network topology of a microgrid system composed of two microgrids and multiple household nodes. The dashed lines represent potential paths for energy distribution or communication between nodes. This topology serves as a graphical representation of the mathematical model, helping to visualize node relationships and system structure. It also plays a key role in formulating and interpreting model constraints, such as energy flow limits, connectivity rules, and operational dependencies between microgrid controllers and household units. By mapping these interactions, the figure supports both the conceptual understanding and the analytical formulation of the optimization problem.

**Fig. 5:**
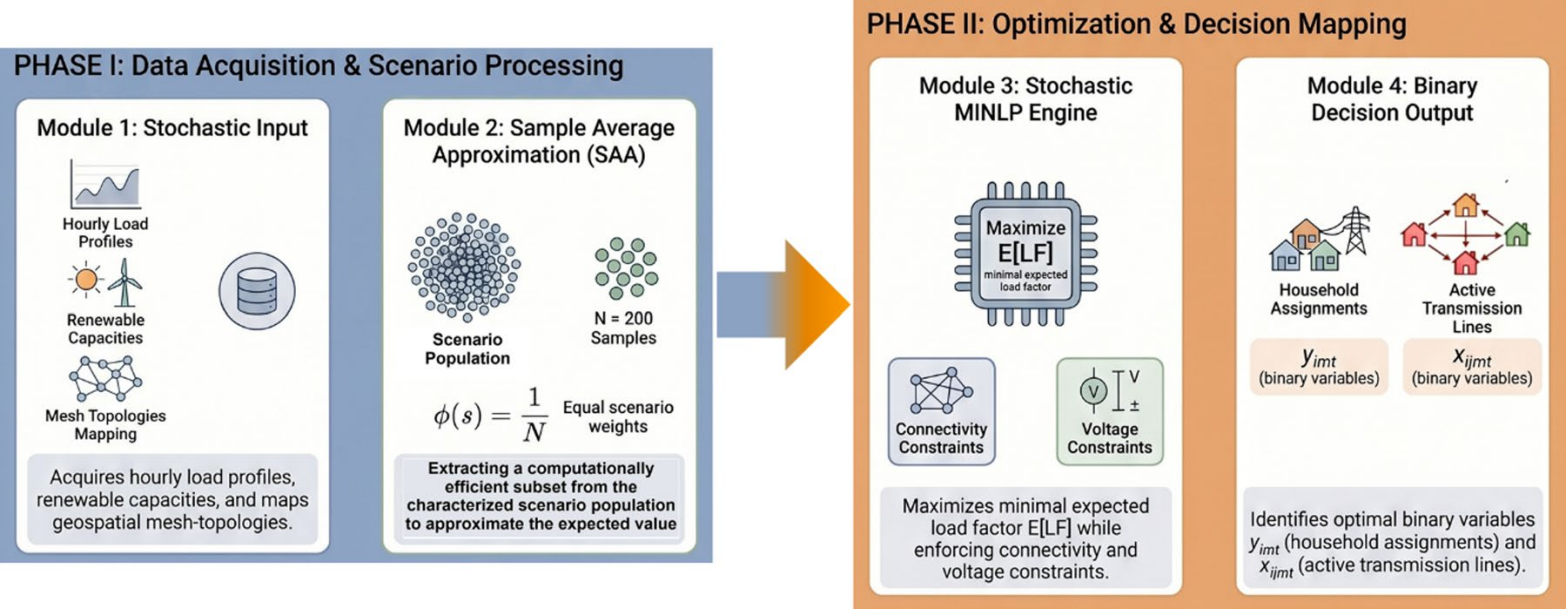
Procedural methodological framework for the stochastic multi-microgrid reconfiguration algorithm.

The procedural execution of the proposed algorithm is visualized in the Methodological Framework (Fig. 5). The framework is structured into four primary modules to ensure mathematical rigor and logical flow. Module 1 manages the acquisition of stochastic demand and renewable profiles and maps them onto the geospatial mesh topology. Module 2 applies SAA processing, in which Monte Carlo sampling reduces the number of realizations to a computationally efficient sample size of *S*. Crucially, each scenario is assigned a probability weight ($\:\varphi\:\left(s\right)$) to approximate the expected value of the load factor. Module 3 represents the Stochastic MINLP engine, which executes the objective function while enforcing physical constraints, including mesh connectivity, flow conservation, and distance-dependent distribution losses. Finally, Module 4 generates the decision output, identifying the optimal hourly binary mappings for household assignments ($\:{y}_{imt}$) and transmission lines ($\:{x}_{ijmt}$), alongside comprehensive performance analytics. While the selection of the optimal sample size (*S*) is justified by the sensitivity analysis presented in Sect.  5.1, the framework establishes the mathematical framework for approximating the expected load factor while enforcing physical network constraints. This framework clarifies the mathematical transition from scenario sampling to robust performance metrics, ensuring physical consistency with network distribution losses^37^.

### Assumptions

To ensure model tractability and technical consistency, the following foundational assumptions are adopted within the proposed framework:

- The microgrids operate without integrating energy storage systems.
- The planning horizon is defined as a single operational day, discretized into 24 one-hour intervals.
- To maintain physical consistency across the optimization framework, all parameters including demand ($\:{D}_{hts}$), generation capacity ($\:{C}_{mts}$), and power consumption ($\:{W}_{mts}$ and $\:{E}_{ms}$) are standardized to kWh.
- Each household must be assigned to exactly one microgrid at each time step, while each microgrid must supply one or more households.
- Power generation in microgrids is derived from a combination of a conventional diesel unit and renewable energy sources like photovoltaic and wind turbines. The output of these renewable units is modeled as a stochastic variable dependent on temporal weather conditions.
- Distribution losses are quantified based on household demand and geospatial location within the mesh topology. These losses are modeled based on the characteristics and vary dynamically with the active routing path.
- A zero-load-shedding policy is implemented to ensure that household consumption patterns remain completely undisturbed. Resilience is achieved through dynamic reconfiguration, allowing households to be rerouted to alternative microgrids during localized supply deficits or microgrid outages.
- Households are modeled as intermediate routing nodes, functioning as smart junctions that partition electrical power between local demand and the supply for neighboring households. This configuration establishes a flexible network topology, facilitating multidirectional power sharing across various distribution paths.
- It is assumed that underlying power electronics and secondary control layers handle the transient synchronization of reconfigurations, allowing this study to focus on the stochastic optimization of energy flows.

The proposed stochastic MINLP mathematical formulation using the SAA method for the problem is presented below with sample size *S*. The time unit is one hour, and the time horizon *T* is one day (1 day = 24 h). The indices, parameters, and decision variables used in this paper are summarized in Table 2.

**Table 2:** Notation summary.

Sets and indices	
$\:H$	Set of households
$\:h$	$\:\mathrm{Index}\text{}\mathrm{for}\text{}\mathrm{households\:}\{\mathrm{1,2},\dots\:..,H\}$
$\:M$	Set of microgrids
$\:m$	$\:\mathrm{Index\:for\:}\mathrm{microgrids\:}\{\mathrm{1,2},\dots\:,M\}$
$\:N$	Set of all nodes, $\:N=M\cup\:H$
$\:T$	Set of time periods
$\:t$	$\:\mathrm{I}\mathrm{ndex\:for\:time\:slot\:}\{\mathrm{1,2},\dots\:,T\}$
$\:i\ and\ j$	$\:\mathrm{Index\:for\:nodes\:in\:the\:network\:}\{\mathrm{1,2},\dots\:,N\}$
$\:DC\left(i\right)$	Set of nodes which are on the path that supply node $\:\left(i\right)$
$\:S$	Sample size or the total number of scenarios for stochastic modelling
$\:s$	$\:\mathrm{Index\:for\:uncertainty\:scenarios}\:\left\{\mathrm{1,2},\dots\:,S\right\}.$
**Parameters**	
$\:{D}_{hts}$	Demand (i.e. energy consumed) of household $\:h$ at time *t* under scenario *s* (kWh).
$\:{C}_{mts}$	Capacity (i.e. total amount of energy produced) of Microgrid $\:m$ at time *t* under scenario *s* (kWh).
$\:{L}_{ijm}$	Resistance of the branch $\:(i-j)$ supplied from microgrid $\:\left(m\right)-{\Omega\:}/m$.
$\:{Tloss}_{s}$	The total power loss over the whole network considering scenario $\:\left(s\right)\:-\:kW$
$\:{\widehat{I}}_{its}$	The current needed for household $\:h$ at time *t* under scenario *s* (Amp).
Decision Variables	
$\:{x}_{ijmt}$A binary decision variable which is equal to 1 if node $\:\left(i\right)$ is connected to node $\:\left(j\right)$ supplied from microgrid $\:\left(m\right)$ at time period $\:\left(t\right)$, and 0 otherwise.	
$\:{y}_{imt}$A binary decision variable which is equal to 1 if node $\:\left(i\right)$ is supplied form microgrid $\:\left(m\right)$ at time period $\:\left(t\right)$, and 0 otherwise.	
**Auxiliary Decision Variables**	
$\:{I}_{imts}$	The total current over the path from microgrid $\:\left(m\right)$ to node $\:\left(i\right)$ at time period $\:\left(t\right)$ considering scenario $\:\left(s\right)-Amp$
$\:{loss}_{mts}$	The energy loss over the path supplied from microgrid $\:\left(m\right)$ at time period $\:\left(t\right)$ considering scenario $\:\left(s\right)-kWh$
$\:{W}_{mts}$	Total energy consumed over the path supplied from microgrid $\:\left(m\right)$ at time period $\:\left(t\right)$ considering scenario $\:\left(s\right)-kWh$
$\:{E}_{ms}$	Total energy consumed over the paths supplied from microgrid $\:\left(m\right)$ over the 24 h considering scenario $\:\left(s\right)-kWh$
$\:{AVG}_{ms}$	Average energy consumed over the path supplied from microgrid $\:\left(m\right)$ considering scenario $\:\left(s\right)-kWh$
$\:{MaxD}_{ms}$	Maximum power demand for microgrid $\:\left(m\right)$ considering scenario $\:\left(s\right)-kW$
$\:{LF}_{ms}$	Load factor for microgrid $\:\left(m\right)$ considering scenario $\:\left(s\right)$

**Objective function**.

The primary objective of the proposed framework is to maximize the minimum expected load factor index across the multi-microgrid network. This formulation is inspired by robust optimization methodologies, which seek to ensure system reliability even under adverse operational scenarios^38^. The load factor (LF) is a critical indicator of power utilization efficiency, comparing the average load over a specific interval to the peak load during that same period. The index is quantitatively improved as its value approaches unity^39^. This hierarchical optimization is achieved through a multi-step stochastic process:

1. *Scenario-based evaluation* The randomness in microgrid and household demand and in microgrid and energy sources capacity is denoted by a scenario *s*. Let $\:\varphi\:\left(s\right)$ be the corresponding probability of scenario *s* ∈ *S*, and $\:\sum\:_{s}\varphi\:\left(s\right)$ =1. A scenario defines the vector of parameters and auxiliary variables, respectively: demand of household *h* ($\:{D}_{hts}$), capacity of microgrid *m* ($\:{C}_{mts}$), the current needed for household *h* ($\:{\widehat{I}}_{its}$), power demand for microgrid *m* ($\:{E}_{ms}$), average power consumed for microgrid *m* ($\:{AVG}_{ms}$), power consumed for microgrid *m* ($\:{W}_{mts}$), maximum power demand for microgrid *m* ($\:{MaxD}_{ms}$), the load factor for microgrid *m* ($\:{LF}_{ms}$) and the total amount of current enters node *i* and passes from microgrid *m* ($\:{I}_{imts}$).
2. *Expectation Estimation* Utilizing the SAA method, the expected LF for each microgrid is estimated by calculating the mean performance across all scenarios. Since the stochastic form of the objective function ($\:\left[\sum\:_{s=1}^{S}\varphi\:\left(s\right)\times\:{LF}_{ms}\right]$) cannot be directly optimized, Monte Carlo sampling is applied to estimate the expected value, with sample size *S*. The expected LF for each microgrid is approximated by calculating the mean performance across a finite sample size of stochastic realizations:

$$\:{\mathbb{E}}_{s}\left[{LF}_{ms}\right]\approx\:\left[\frac{\:1\:}{\:S\:}\:\bullet\:\sum\:_{s=1}^{S}{LF}_{ms}\right]\:$$

3. *Max-Min Optimization* The model identifies the microgrid with the minimal expected LF (the worst-performing unit in terms of average efficiency) and targets the maximization of this value.

$$\:\boldsymbol{M}\boldsymbol{a}\boldsymbol{x}\boldsymbol{i}\boldsymbol{m}\boldsymbol{i}\boldsymbol{z}\boldsymbol{e \ }Z=\underset{m}{\mathrm{Min}}\left({\mathbb{E}}_{s}\left[{LF}_{ms}\right]\right)\approx\:\underset{m}{\mathrm{Min}}\left(\left[\frac{\:1\:}{\:S\:}\:\bullet\:\sum\:_{s=1}^{S}{LF}_{ms}\right]\right)$$

This robust max-min formulation ensures that the optimization strategy prioritizes the most constrained microgrid in the network. By elevating the performance of the weakest microgrid, the framework generates a robust solution that ensures system-wide operational stability and effective peak-load leveling, even under worst-case fluctuations in demand and supply. This architectural strategy effectively smooths load variations across the mesh topology, creating a distributed buffer that enhances overall system resilience.

**Constraints**.

**Table:** 

$\:\sum\:_{j\in\:H}{x}_{ijmt}\ge\:1$	$\:\forall\:\:i\in\:H\:m\in\:M\:|\:m=i\:t\in\:T\:$	(1)
$\:\sum\:_{i\in\:N}\sum\:_{m\in\:M}{x}_{ijmt}=1$	$\:\forall\:\:j\in\:H,\:t\in\:T$	(2)
$\:{x}_{ijmt}+{x}_{jimt}\le\:1$	$\:\forall\:\:i\in\:N\:j\in\:N\:m\in\:M\:t\in\:T$	(3)
$\:\sum\:_{m\in\:M}{y}_{imt}=1$	$\:\forall\:\:i\in\:H\:t\in\:T$	(4)
$\:\sum\:_{i\in\:N}{x}_{ijmt}={y}_{jmt}$	$\:\forall\:\:j\in\:H\:m\in\:M\:t\in\:T$	(5)
$$\begin{aligned} I_{{jmts}} = & \hat{I}_{{jts}} + \sum {_{{i \in DC\left( j \right)}}^{{}} } \hat{I}_{{its}} \times x_{{jimt}} \\ & + \sum {_{{i \in DC\left( j \right)}} } \sum {_{{k \ne i \in DC\left( j \right)}} } \hat{I}_{{kts}} \times x_{{ikmt}} \\ \end{aligned}$$	$\:\forall\:\:j\in\:H\:m\in\:M\:t\in\:T\:s\in\:S$	(6)
$\:\sum\:_{i\in\:N}^{}\sum\:_{j\in\:H}^{}{x}_{ijmt}\times\:{L}_{ijm}\times\:{I}_{jmts}^{2}={loss}_{mts}$	$\:\forall\:\:m\in\:M\:t\in\:T\:s\in\:S$	(7)
$\:\sum\:_{m\in\:M}^{}\sum\:_{t\in\:T}^{}{loss}_{mts}\le\:{Tloss}_{s}$	$\:\forall\:\:s\in\:S$	(8)
$\:{loss}_{mts}+\sum\:_{i\in\:H}^{}{D}_{its}\times\:{y}_{imt}={W}_{mts}$	$\:\forall\:\:m\in\:M\:t\in\:T\:s\in\:S$	(9)
$\:\sum\:_{t\in\:T}^{}{W}_{mts}={E}_{ms}$	$\:\forall\:\:m\in\:M\:s\in\:S$	(10)
$\:{W}_{mts}\le\:{C}_{mts}$	$\:\forall\:\:m\in\:M\:t\in\:T\:s\in\:S$	(11)
$\:{AVG}_{ms}={E}_{ms}/\left(T\right)$	$\:\forall\:\:m\in\:M\:s\in\:S$	(12)
$\:{{MaxD}_{ms}\ge\:W}_{mts}$	$\:\forall\:\:m\in\:M\:t\in\:T\:s\in\:S$	(13)
$\:{LF}_{ms}={AVG}_{ms}/{MaxD}_{ms}$	$\:\forall\:\:m\in\:M\:s\in\:S$	(14)
$\:{y}_{imt},$ $\:{x}_{ijmt}$∈ {0, 1}	∀ i $\:\in\:N,\:j\in\:N,$ m ∈ M, t ∈ T	(15)
$\:{LF}_{ms},{E}_{ms},\:{W}_{mts},{AVG}_{ms},{Max D}_{ms},{I}_{jmts}\ge\:0$	∀ i $\:\in\:N,\:j\in\:N,\:$m ∈ M, t ∈ T, s ∈ S	(16)

**Fig. 6:**
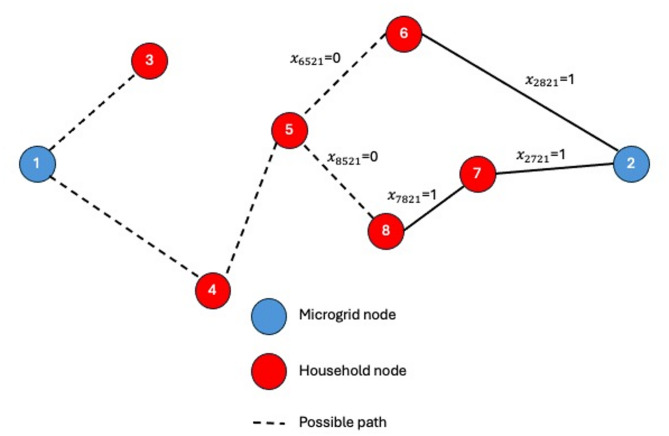
Constraint (2) explanation.

Constraint (1) guarantees that each microgrid must be connected to at least one household. Constraint (2) ensures that if a household to be supplied from a microgrid, at most one arc should be activated to this node. As shown in Fig. 6, considering the first time period, node (5) has two options to be supplied from microgrid (2), either via node (6) or (8), and one option to be supplied from microgrid (1), which is via node 4The constraint ensures that only one arc is chosen for node (5), either $\:{x}_{8521}$, $\:{x}_{6521},$ or $\:{x}_{4511}$.

**Fig. 7:**
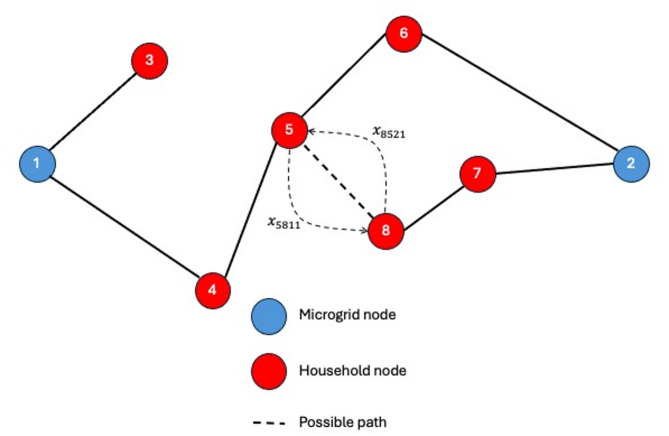
Constraint (3) explanation.

Constraint (3) ensures that no bidirectional supply (i.e. cyclic supply) is allowed between the households. As shown in Fig. 7, either node (5) to supply node (8) or node (8) to supply node (5), therefore $\:{x}_{5811}+{x}_{8521}\le\:1$.

**Fig. 8:**
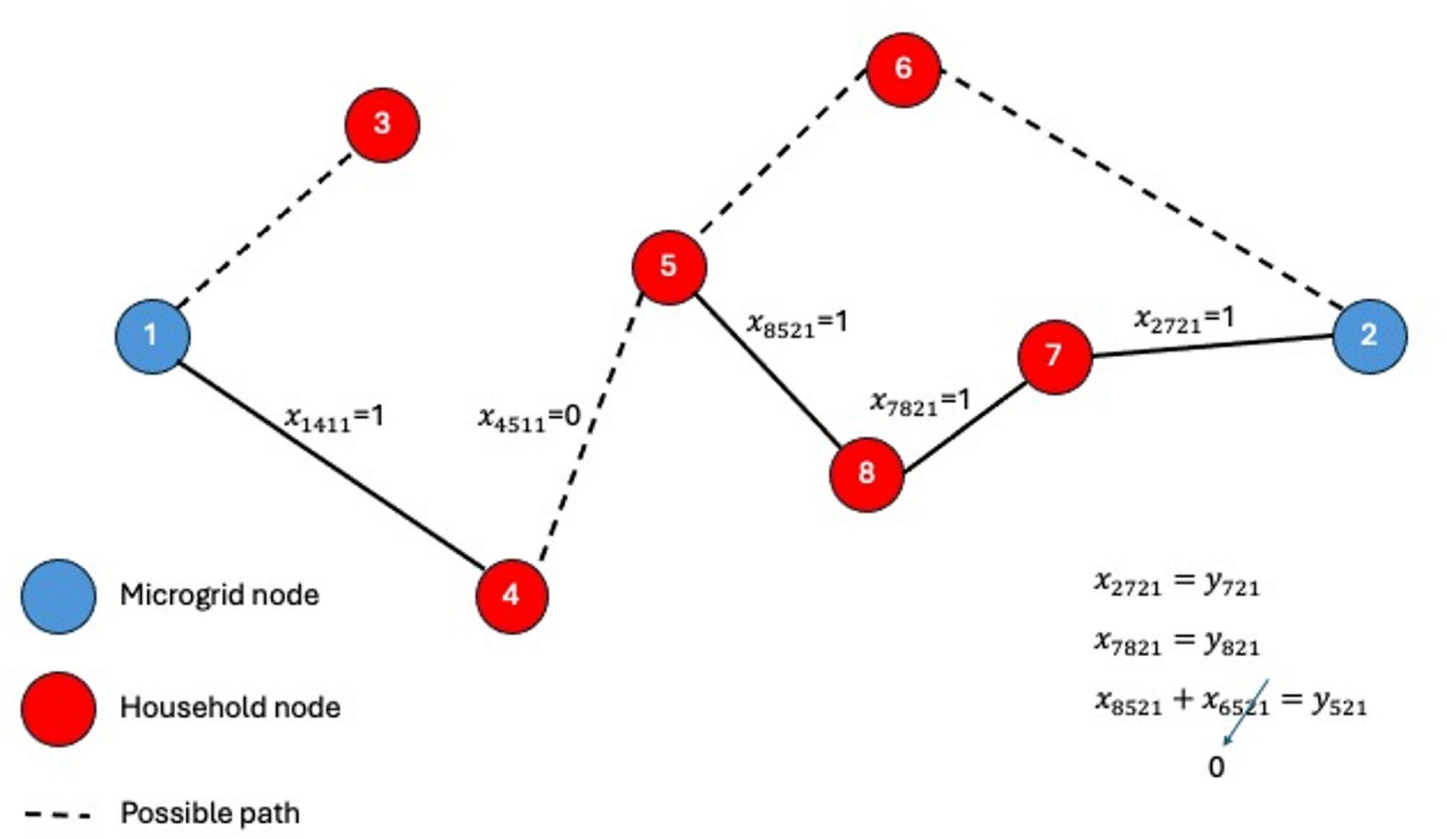
Constraint (5) explanation.

Constraint (4) prevents any household at any time period from being supplied by more than a microgrid. Constraint (5) ensures that if a node is to be supplied from a microgrid, the path from the microgrid to the destination node is interconnected. As shown in Fig. 8, if node (5) is to be supplied by microgrid (2), then the path from the microgrid to node (5) should be interconnected. In this example node (7) is directly connected to microgrid (2), node (8) is connected to microgrid (2) through node (7), and node (5) is connected to microgrid (2) through nodes (8) and (7).

**Fig. 9:**
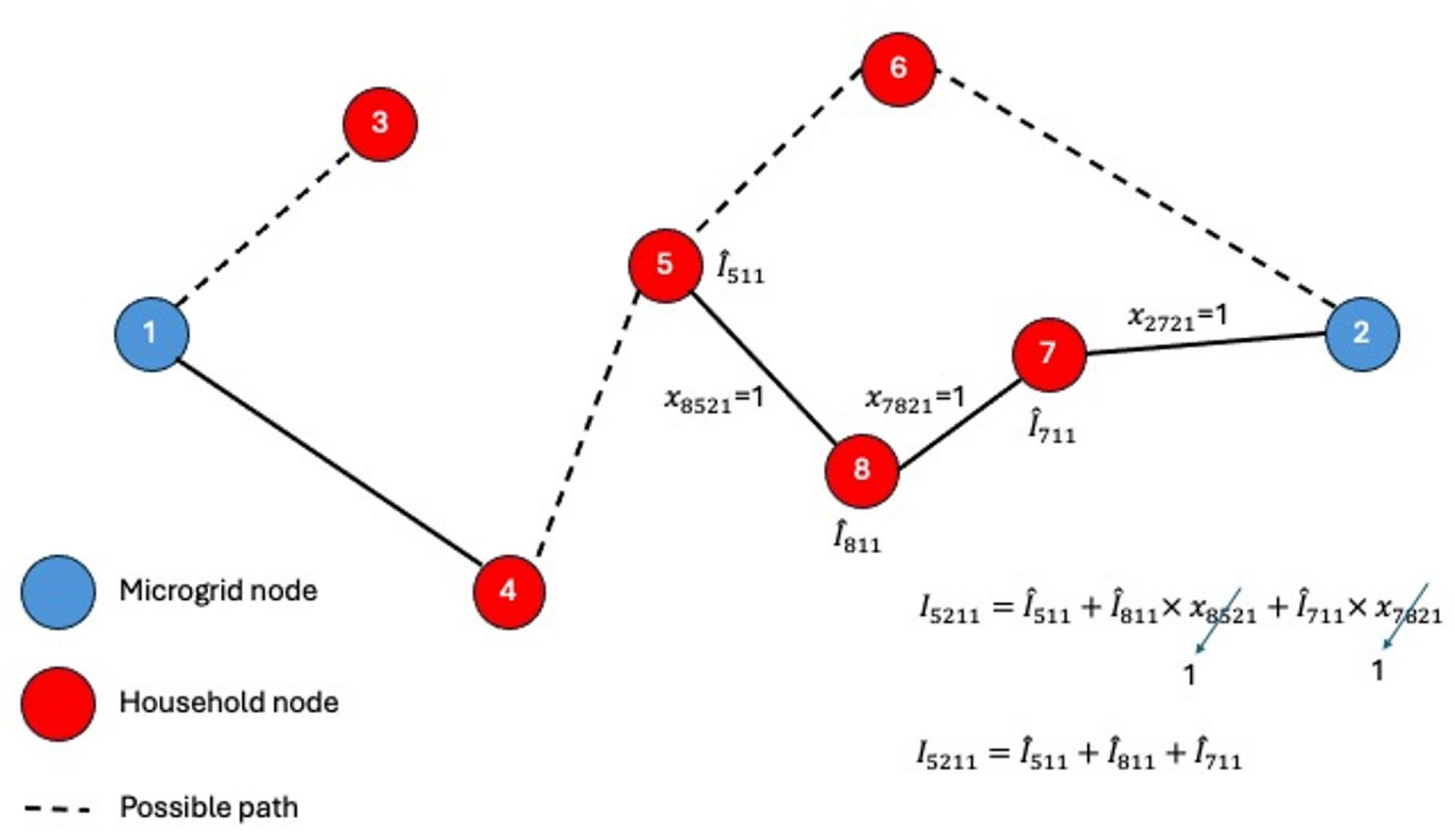
Constraint (6) explanation.

Constraint (6) is used to calculate the total current over the path from microgrid $\:\left(m\right)$ to household $\:\left(j\right)$ at time $\:\left(t\right)$ considering scenario $\:\left(s\right)$ $\:\left(Amp\right)$. The total current over the path from the microgrid $\:\left(m\right)$ to household $\:\left(j\right)$ is the current needed by node $\:\left(j\right)$ in addition to the current of the nodes that precedes node $\:\left(j\right)$ in the path. This constraint also works as a flow conservation constraint. Figure 9 shows the working mechanism of constraint (6) for the path from microgrid (2) to household (5). If household (5) is to be supplied from microgrid (2) via the pass 2-7-8-5, then the total current till node (5) is equal to the current at node (5) plus the current at the nodes that are on the path (i.e. nodes 8 and 7). First, the constraint calculates the current at the destination node (node 5), then it adds the current of the node that is directly connect to the destination node (i.e. node 8). In the same way, the current of node (7) is added to the total current.

Constraint (7) calculates the loss for each microgrid $\:\left(m\right)$ at time period $\:\left(t\right)$ for a given scenario $\:\left(s\right)$. Constraint (8) limits the total loss for a given scenario to be less than a threshold value. Constraint (9) calculates the total energy required to be supplied from a microgrid $\:\left(m\right)$ at time period $\:\left(t\right)$ for a given scenario $\:\left(s\right)$. Total energy equals the total demand for the households supplied from the microgrid plus the losses along the path connected to the microgrid. Constraint (10) determines the total energy for microgrid $\:\left(m\right)$ at scenario $\:\left(s\right)$, and constraint (11) limits the total energy supplied from the microgrid $\:\left(m\right)$ at time period $\:\left(t\right)$ considering scenario $\:\left(s\right)$ to be less than a given capacity of microgrid at specific time period given a specific scenario.

Constraint (12) determines the average power supplied from microgrid $\:\left(m\right)$ considering scenario $\:\left(s\right)$, constraint (13) calculates the maximum power generated from microgrid $\:\left(m\right)$ given scenario $\:\left(s\right)$. And finally, constraint (14) calculates the load factor for microgrid $\:\left(m\right)$ given a specific scenario $\:\left(s\right)$.

Finally, constraints (15) and (16) are binary integer and non-negativity constraints for decision variables.

## Case study and data set

This study validates the proposed stochastic dynamic reconfiguration model using a synthesized, harmonized dataset constructed from multiple public sources to represent a realistic and challenging MMG network. The core data comprises hourly load profiles for 15 distinct households and generation profiles for three heterogeneous microgrids, each with a unique mix of renewable and conventional energy sources. As the raw data originated from disparate sources, a critical harmonization and scaling process was undertaken to ensure dimensional consistency and operational realism within a unified simulation environment. A key feature of the proposed model is the explicit incorporation of distance-dependent power loss between households and microgrids, a critical factor often abstracted in related works. The subsequent subsections detail the network topology, the characterized demand uncertainties, and the supply-side generation profiles that form the basis of the conducted numerical analysis.

### Network configuration and distance-based losses

The network under study is a mesh-topology-based multi-microgrid system supplying power to 15 households. This advanced configuration is a key differentiator of this study, as it enables dynamic, redundant pathways for power flow. Unlike traditional radial distribution networks, where each load is supplied by a single source through a unique path, the mesh network enables any household to be supplied by any microgrid through multiple potential routes, significantly enhancing system reliability, resilience, and operational flexibility.

**Fig. 10:**
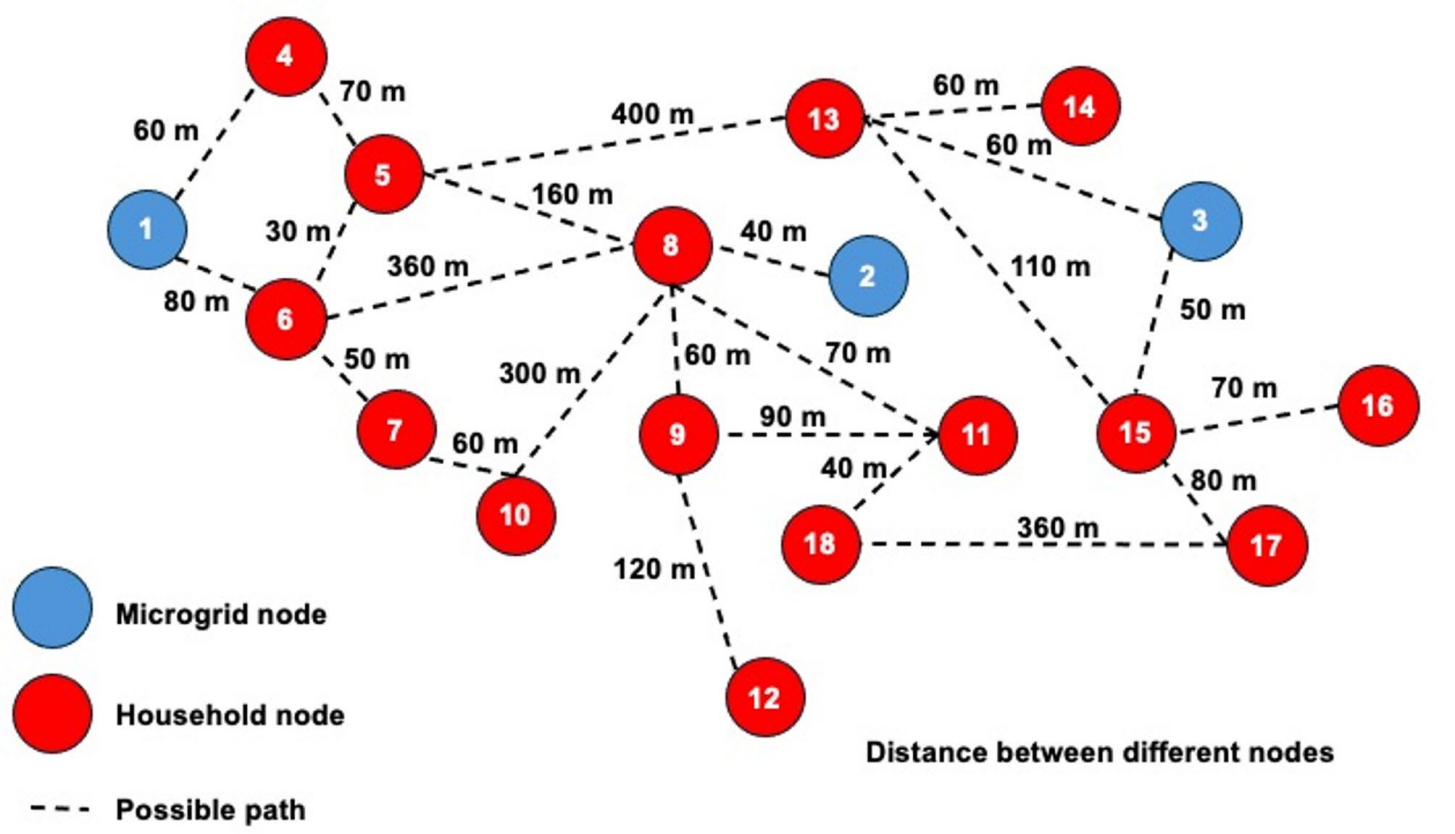
Network topology of the three-microgrid system serving 15 households, with distances annotated.

The topological configuration, including all possible interconnections (edges) between nodes (households and microgrids) and the Euclidean distances between them, is illustrated in Fig. 10. These distances, adopted from the work of Ismail et al.^15^, ground the conducted analysis in a physically realistic geospatial layout and are a primary input to the loss function. The network comprises three distinct microgrids:

- Microgrid 1 (MG1): Comprises an off-shore wind turbine and a diesel generation unit.
- Microgrid 2 (MG2): Comprises an on-shore wind turbine and a diesel generation unit.
- Microgrid 3 (MG3): Comprises a PV power unit and a diesel generation unit.

In this mesh network, the dynamic reconfiguration problem involves determining the optimal pathways and power flows from the source microgrids to the sink households. The topology reveals significant spatial variety and numerous routing options. For instance, Household 10 can be supplied by MG2 via Household 8 (total distance of 340 m) but can also be reached via a shorter path from MG1 through the route Household 6 → Household 7 (total distance of 190 m). Similarly, Household 5 exemplifies high connectivity, as it can be powered by any of the three microgrids: from MG1 via Household 4 or 6, from MG2 via Household 8, or from MG3 via Household 13. The abundance of feasible pathways creates a complex optimization landscape in which the algorithm must evaluate numerous non-trivial routing options to minimize losses and cost, or to maximize the load factor index, as demonstrated in this study.

Consequently, each household can be supplied by any microgrid through a multitude of paths, with other households acting as intermediate routing nodes. Consequently, each household can be supplied by any microgrid through a multitude of paths, with other households acting as intermediate routing nodes. In this case study, each household is assumed to be connected to a distribution junction within the microgrid network. At this junction, the electrical current can be divided into two paths: one supplying the local household load and the other continuing through the distribution line to supply neighboring households. This configuration allows households to be represented as interconnected nodes within the microgrid, enabling the distribution and transfer of electricity among residential units. This architecture creates a highly interconnected and resilient grid. For example, if a primary path to a critical load is compromised, supply can be maintained through an alternative route; power from MG2 could still reach Household 6 via Household 8 even if a direct connection with MG1 fails. This redundancy ensures that the network can dynamically adapt to maintain service, leveraging the mesh topology to enhance reliability beyond the capabilities of a radial system.

Power loss during transmission is modeled as a function of the cumulative distances along the entire chosen pathway. This model imposes a crucial spatial cost element, making the trade-off between using a longer, available path versus a shorter, potentially congested one a central part of the optimization. The objective is to find configurations that not only balance supply and demand but also minimize the total system losses incurred across these distances, a task for which the mesh topology provides essential flexibility.

### Characterization of demand uncertainty

**Fig. 11:**
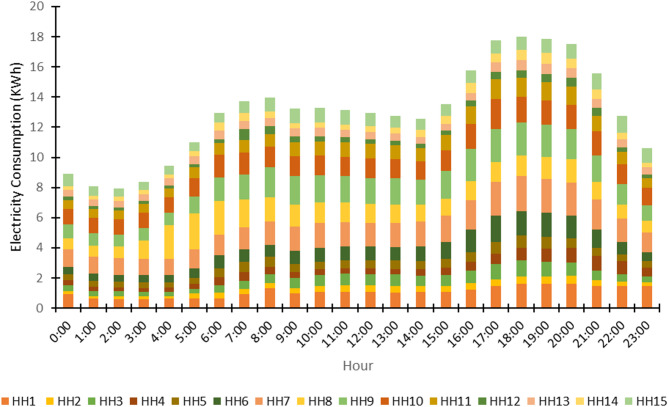
Demand patterns - Average hourly load consumption profile for a typical day.

The raw load data for the 15 households was sourced from many public repositories. The average hourly load consumption profile for a typical day, aggregated across all households, is presented in Fig. 11, showing characteristic peaks and troughs aligned with daily human activity.

**Fig. 12:**
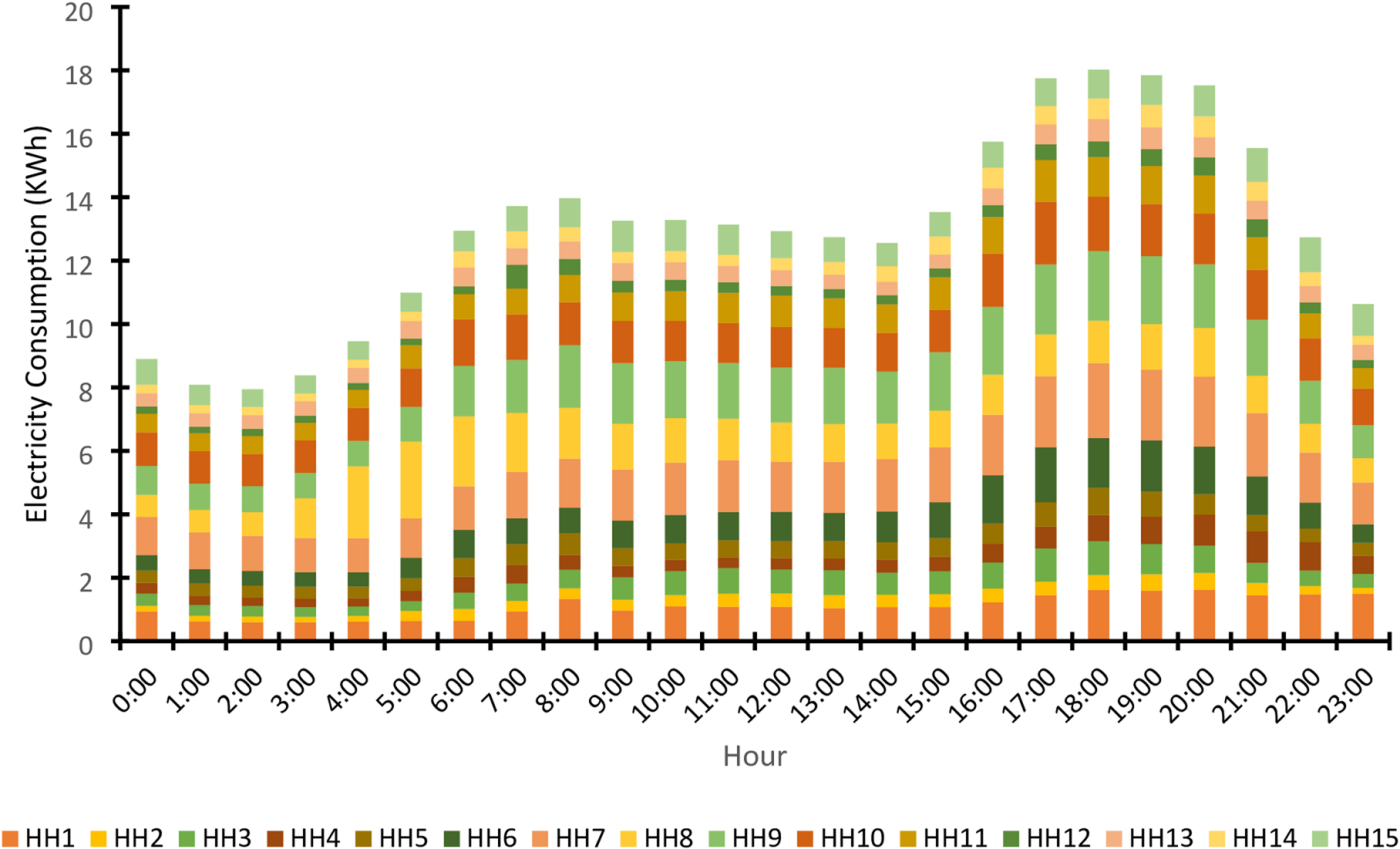
Demand patterns -Box plots representing the variation of the aggregated load across 600 scenarios for each hour of the day.

To capture the inherent uncertainty in electricity demand, 600 scenarios of hourly load patterns over a 24-hour period are considered and analyzed. These scenarios account for variability between households and stochastic deviations from the average profile. Figure 12 presents box plots of the aggregated hourly consumption across all 600 scenarios. The plots effectively visualize the interquartile range, the median, and outliers of the forecast uncertainty at each hour, providing a robust basis for the proposed stochastic optimization model. The significant variation, particularly during peak hours, underscores the necessity of a probabilistic approach to ensure network reliability.

The average hourly load profiles for the 15 households (Fig. 11) reveal significant heterogeneity in consumption patterns, with varying magnitudes, peak timings, and curve shapes indicative of diverse occupant behaviors and appliance usage. This spatial and temporal diversity in demand is a fundamental driver for the proposed dynamic reconfiguration model, as it enables the mesh network to optimize power flow by strategically matching microgrid supply with clustered demand patterns, thereby improving efficiency and reducing losses through intelligent routing.

### Characterization of supply-side generation

**Fig. 13:**
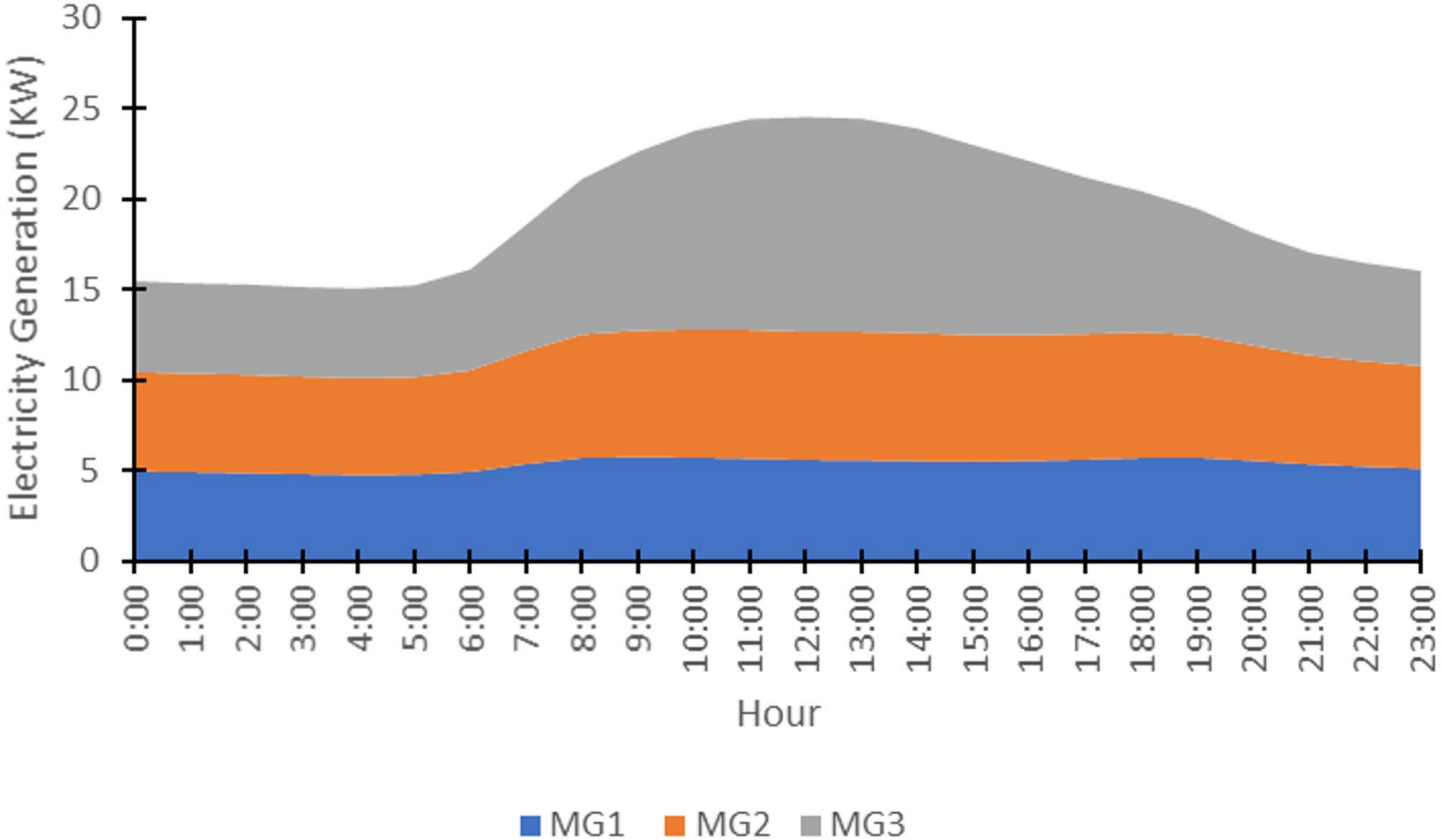
Supply-side capacity - Average hourly generation capacity profiles for MG1, MG2, and MG3.

The generation capacities of the renewable sources in each microgrid were scaled to be commensurate with the aggregated load demand after harmonization. The average hourly capacity for each of the three microgrids is shown in Fig. 13, highlighting the complementary nature of the generation profiles: MG3’s solar generation peaks during midday, while the wind generation in MG1 and MG2 exhibits a different pattern.

**Fig. 14:**
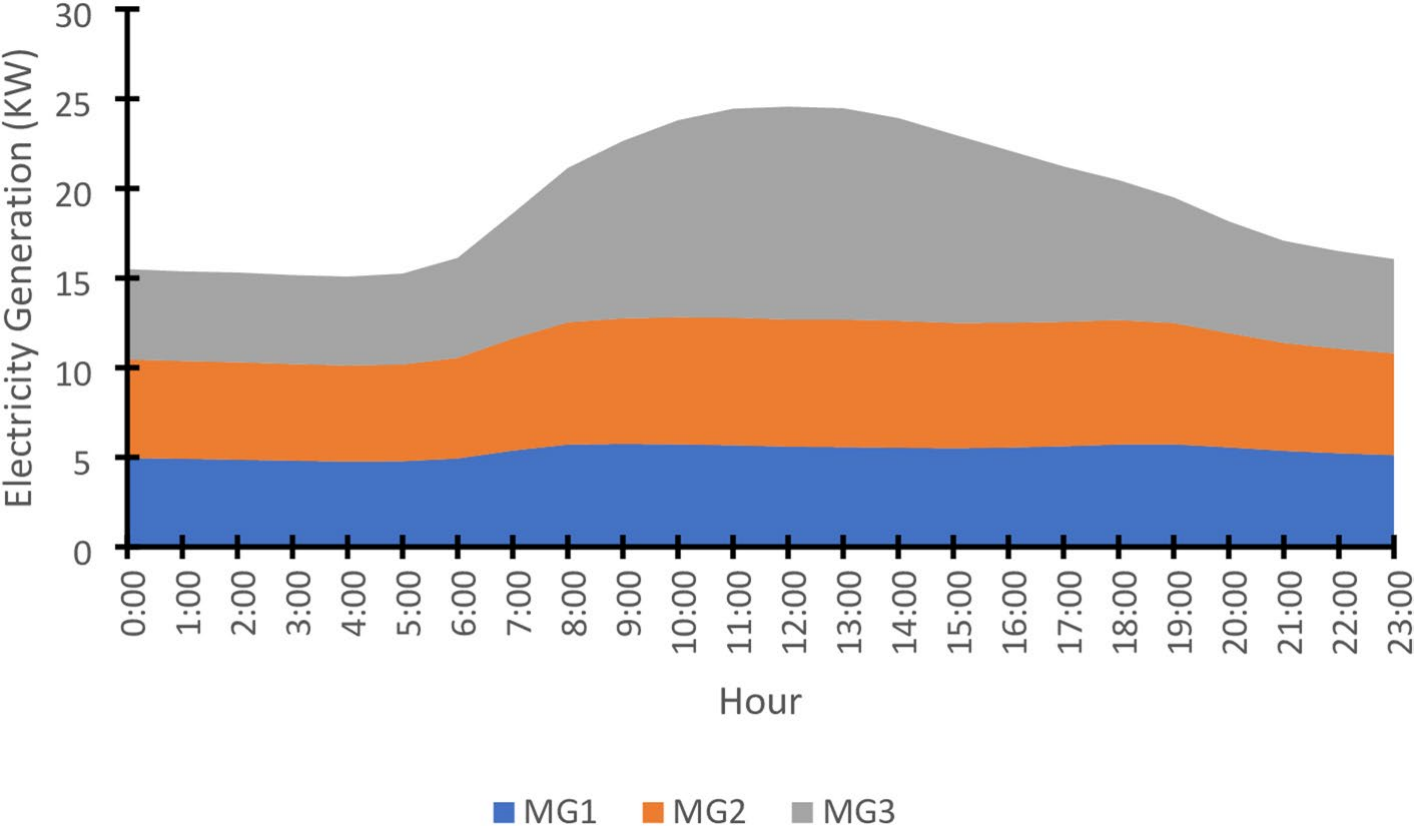
Supply-side capacity - Box plots representing the variation of the aggregated capacity profiles for MG1, MG2, and MG3 across 600 scenarios for each hour of the day.

Like the demand side, the renewable generation is subject to uncertainties. Box plots for each microgrid’s capacity (Fig. 14) illustrate the hourly variability across the 600 scenarios, stemming from uncertainties in wind speed and solar irradiance. This comprehensive representation of both demand and supply uncertainties is fundamental to the stochastic framework of the proposed optimization model.

The average hourly generation capacity for the three microgrids is presented in Fig. 13, highlighting their complementary generation profiles. MG3’s solar-based output peaks predictably during midday hours, while the wind-powered generation of MG1 (off-shore) and MG2 (onshore) exhibits distinct temporal patterns that differ from each other and from the solar profile. This diversity in supply characteristics is crucial for stochastic optimization, as it allows the dynamic reconfiguration model to leverage the strengths of each source—using solar abundance during peak sun hours and harnessing available wind power at other times—to balance the heterogeneous demand efficiently across the mesh network.

The temporal dynamics of the average hourly generation capacity (Fig. 13) provide a deterministic view of supply trends, highlighting the complementary nature of microgrids. However, this box plot (Fig. 14) reveals the profound stochastic reality underlying these averages, quantifying the significant uncertainty in electricity generation capacity across a 24-hour period, characterized by a significant daytime peak due to substantial solar contribution, directly informs the stochastic optimization model. The substantial interquartile ranges and whiskers demonstrate that the actual available capacity at any given time can deviate considerably from the average value. The algorithm must dynamically reconfigure the mesh network not only to match local demand with the most suitable source but also to manage the broader challenge of allocating this time-varying total generation capacity efficiently across the 15 heterogeneous households to minimize overall cost and loss.

In summary, the case study is characterized by a mesh network that offers rich reconfiguration possibilities, significant demand heterogeneity, and generation portfolios with complementary yet uncertain profiles. This combination creates a complex stochastic optimization problem where the objective is the maximization of the minimum expected load factor by dynamically managing power flow across uncertain scenarios. An empirical probability distribution derived from historical data is utilized within the SAA framework. This non-parametric approach is adopted to avoid potential biases associated with assuming specific distributions (e.g., Normal or Weibull), thereby preserving the spatio-temporal correlations between load and renewable generation across scenarios. These scenarios constitute a discrete approximation of the underlying uncertainty. By sampling directly from two years of historical hourly data, authentic statistical dependencies and ‘fat-tail’ events observed in real-world residential load and renewable generation are accounted for, which are frequently obscured by parametric models. The scenarios were generated through random sampling from the historical dataset to ensure an unbiased representation of the uncertainty space. The following section details the proposed model’s performance in navigating this complexity and compares its efficacy across different scenarios.

## Numerical experiments and results

In this section, the implementation of the proposed mathematical model to a case study is presented. It presents numerical experiments and analytical evaluations conducted to assess the performance and effectiveness of the proposed stochastic SAA model. Three sets of analyses were performed. First, a sensitivity analysis on the number of scenarios (sample size) was carried out to identify the optimal number of scenarios for model implementation and to examine the trade-off between sample size ($\:S$) and solution quality. Second, the dynamic behavior of the proposed system was investigated by solving the 3-MG–15-household configuration over a 24-hour time horizon, with graphical representations illustrating the hourly operational configurations and comparative performance among the three MGs. Third, the case study introduced earlier was examined under three different structural settings—three interconnected microgrids (MG1, MG2, MG3), two microgrids (MG1, MG2), and a single microgrid (MG2). In this final analysis, the LF values were recorded and subjected to statistical analysis and comparison across the configurations to evaluate the impact of interconnection on system performance.

### Number of scenarios experiments

**Fig. 15:**
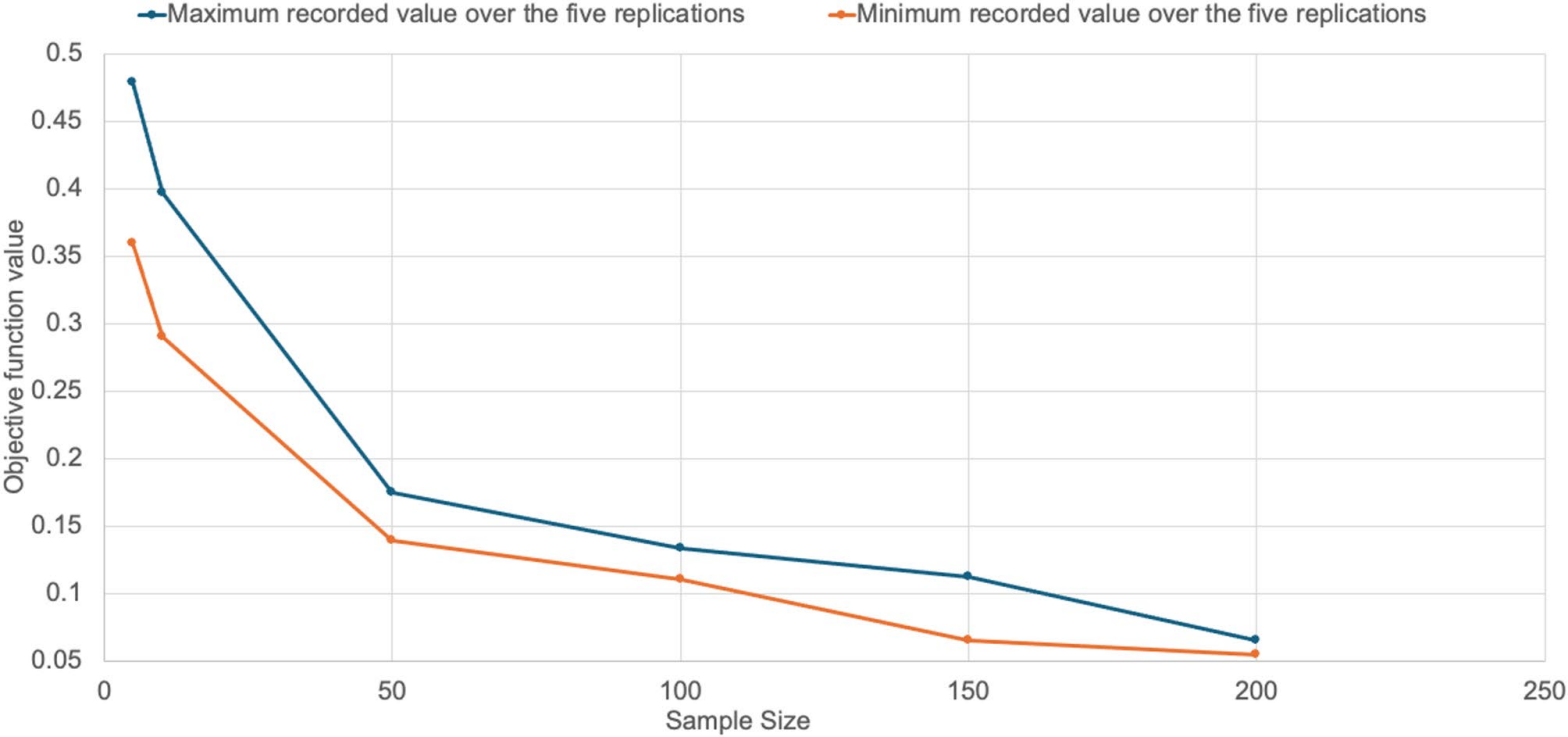
Maximum and minimum recorded value of the objective function over the five replications.

A sensitivity analysis of the “number of scenarios” was conducted to determine the optimal number to use during implementation. This analysis evaluates the trade-off existing between sample size (*S*) and solution quality. The problem was solved using different sample sizes (*S* = 5, 10, 50, 100, 150, and 200), with five replications conducted for each sample size. The highest and lowest values of the objective function across the five replications were recorded for each sample size. The results are tabulated in Table 3. Figure 15 shows the changes of the objective function value with the different sample sizes, specifically, it plots the upper and lower values. Generally, the analysis shows that as the sample size increases, the sample standard deviation decreases, highlighting a more robust solution to the problem, therefore (*S* = 200) was selected for the case implementation.

**Table 3:** The objective function values over the five replications.

Sample Size (S)	Replications					Upper bound	Lower bound	Sample Standard deviation
	1	2	3	4	5			
*S* = 5	0.479	0.422	0.438	0.3598	0.4057	0.479	0.3598	4.36%
*S* = 10	0.39	0.397	0.3082	0.2902	0.297	0.397	0.2902	5.25%
*S* = 50	0.1742	0.166	0.1395	0.1598	0.156	0.1742	0.1395	1.29%
*S* = 100	0.133	0.1248	0.1209	0.1173	0.1102	0.133	0.1102	0.84%
*S* = 150	0.111	0.089	0.065	0.11205	0.08948	0.11205	0.065	1.93%
*S* = 200	0.0596	0.05639	0.06485	0.05395	0.0597	0.06485	0.05395	0.06%

### Number of scenarios experiments

**Fig. 16:**
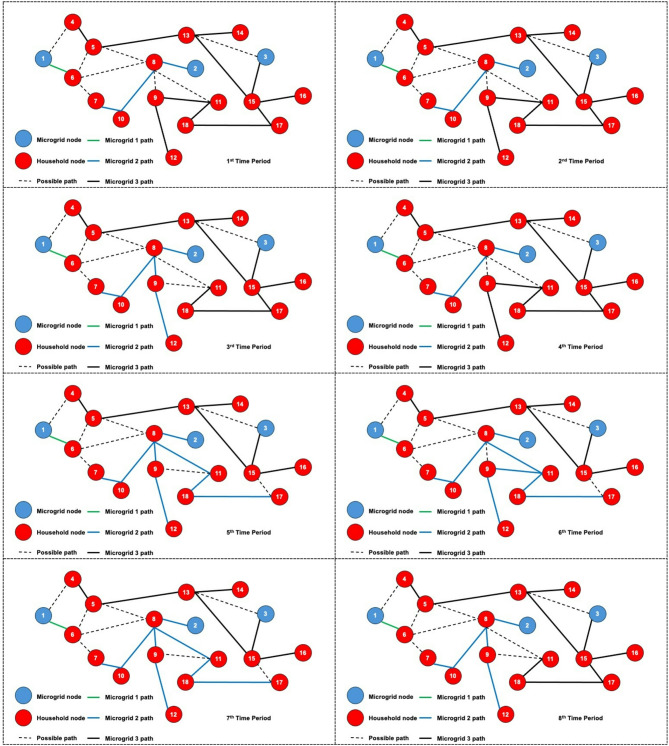
Network configuration for time periods (1 to 8) considering 200 scenarios and 3 microgrids.

**Fig. 17:**
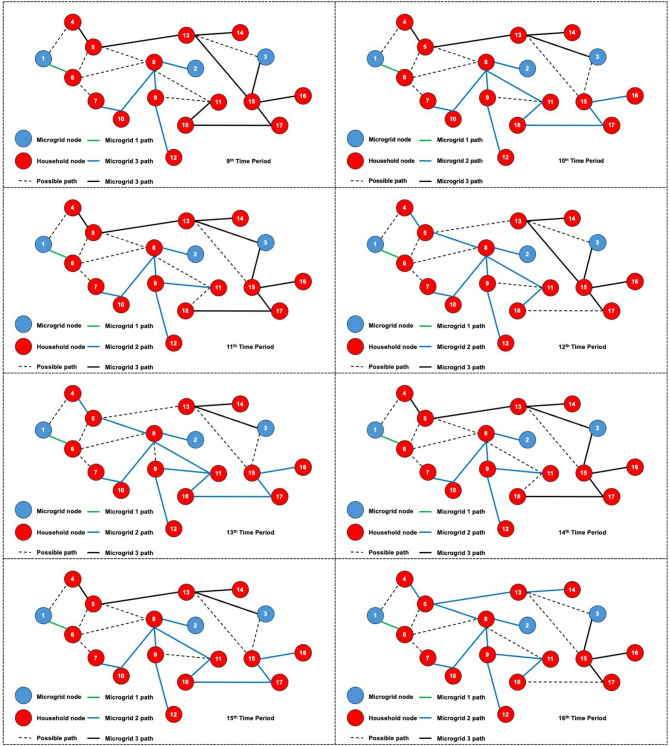
Network configuration for time periods (9 to 16) considering 200 scenarios and 3 microgrids.

**Fig. 18:**
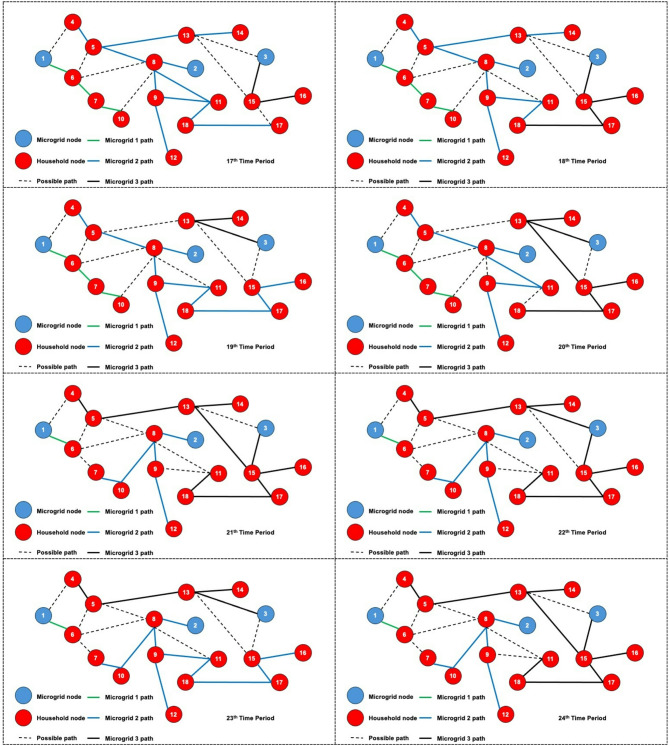
Network configuration for time periods (17 to 24) considering 200 scenarios and 3 microgrids.

**Fig. 19:**
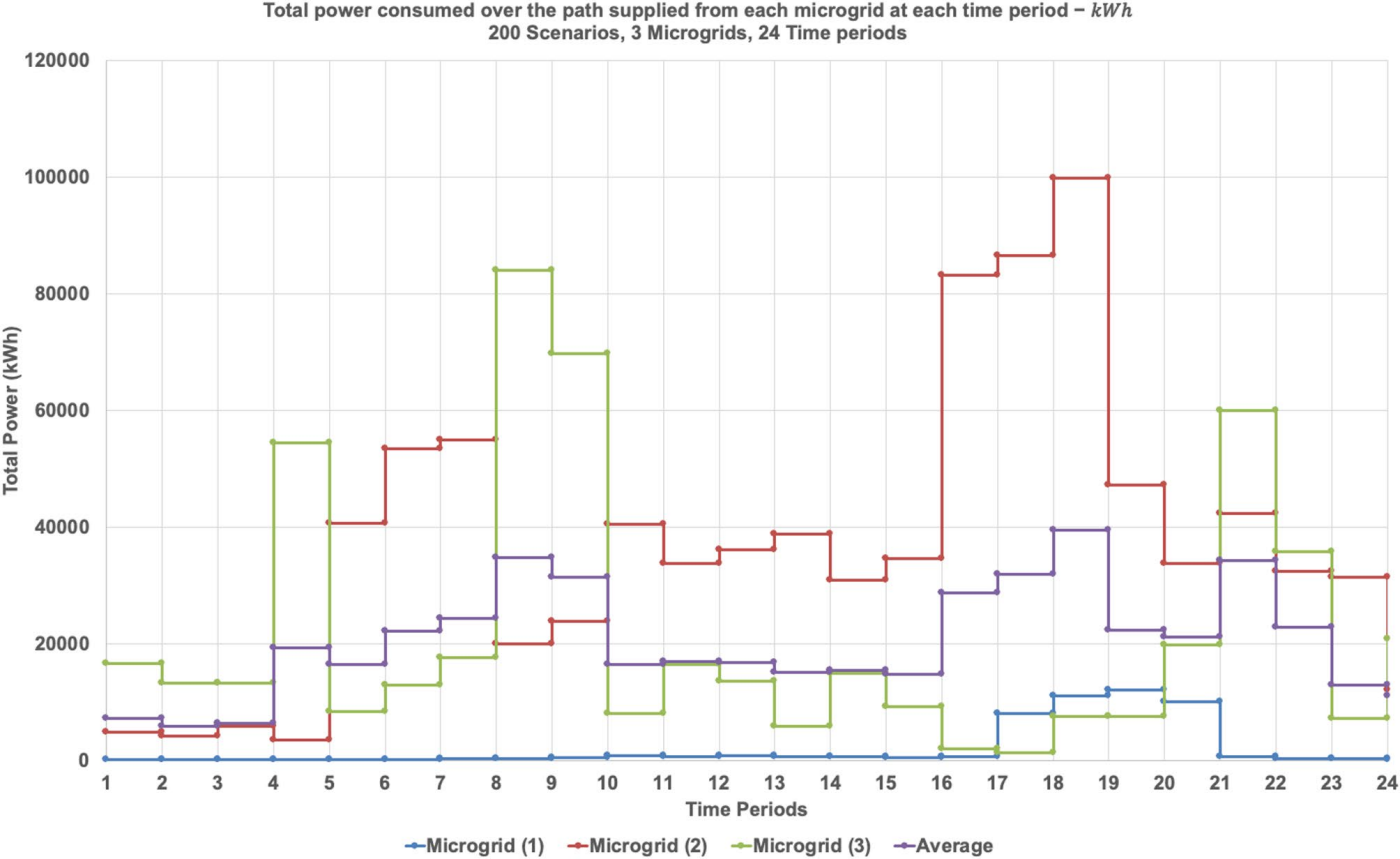
Total power consumed over each path supplied from each microgrid at each time period considering three microgrids (1, 2, and 3) − ℎ.

The dynamic behavior of the proposed model is investigated by solving the 3-MG–15-household configuration over a 24-hour time horizon. The hourly configurations of the system over the 24-hour period are graphically illustrated (Figs. 16, 17 and 18) to demonstrate the temporal variations in microgrid operation. Subsequently, the load factor values for each of the three MGs were computed, with the minimum, maximum, and average values summarized in Table 4. In addition, Fig. 19 presents the total power consumption of each MG over the 24-hour horizon, providing a comparative view of their energy usage patterns.

The network configurations of one of the replications (replication 3) are shown in Figs. 16 and 17, and 18, which are illustrated for time periods (1 to 8), (9 to 16), and (17 to 24), respectively. The results reveal distinct operational roles for the three microgrids and demonstrate the system’s capability to adapt to temporal variations in demand and network conditions.

Microgrid 1 (MG1), represented by green paths, maintains a consistent, stable connection with Household 6 for most of the 24-hour period. During the midday peak hours (periods 17–20), MG1 temporarily extends its service to additional households, indicating a controlled redistribution of supply to alleviate network stress under elevated load conditions.

In contrast, Microgrids 2 and 3 (MG2 in blue paths and MG3 in black paths) display highly adaptive routing behavior, frequently reconfiguring their connections in response to time-dependent demand fluctuations and optimization outcomes. Their operational flexibility underscores their role as dynamic distributors that balance supply among households while responding to stochastic variations in load and resource availability. The observed routing transitions suggest that spatial proximity, temporal load shifts, and system constraints jointly influence the allocation decisions generated by the optimization framework.

The system undergoes noticeable reconfiguration during periods of elevated demand, particularly between hours 17 and 20. The reconfiguration patterns observed in Figs. 16, 17 and 18 demonstrate the model’s inherent sensitivity to distribution losses during the optimization process. All three microgrids are observed to adjust their routing structures by activating previously idle paths and redistributing energy flows to maintain network balance. It is noted that households are predominantly reconnected to adjacent microgrids; the optimizer is shown to bypass more distant nodes even when surplus capacity is available. This behavior is attributed to the losses associated with longer transmission pathways, which would effectively reduce the expected system load factor. Consequently, the model’s ability to incorporate time-dependent constraints while achieving performance-oriented objectives—specifically, minimizing transmission losses and maximizing the minimum expected load factor—is clearly demonstrated.

Dynamic adjustment to fluctuating system conditions is facilitated by an adaptive path-selection mechanism, ensuring an optimal trade-off between reliability and efficiency. By evaluating multiple routing alternatives—represented as potential interconnections within the network topology—the most efficient configuration is selected for each operational time step. The observed temporal evolution of the network validates the robustness of the proposed stochastic model and demonstrates its applicability to real-world multi-microgrid coordination and energy management scenarios. This adaptive behavior confirms the framework’s ability to maintain system stability by proactively rerouting power flows in response to realized demand and supply uncertainties.

Table 4 presents the load factor statistics for each of the three microgrids over the 24-hour period, offering quantitative validation of the behavioral patterns observed in the network configuration figures. Microgrid 1, which was shown to maintain a stable and largely exclusive connection to Household 6, exhibits the lowest average load factor (0.0880), reflecting its role as a dedicated supplier with minimal redistribution. In contrast, Microgrids 2 and 3 demonstrate higher average load factors—0.1405 and 0.1237, respectively—consistent with their dynamic routing behavior and broader service coverage across multiple households. The higher maximum values for MG 2 (0.2177) and MG 3 (0.2257) further indicate their responsiveness during peak-demand periods, particularly between hours 17 and 20, when routing reconfigurations were most pronounced.

**Table 4:** Load factor values over the 200 scenarios considering different settings.

Microgrid	Max load factor value	Min load factor value	Average load factor value
1	0.1355	0.0648	0.0880
2	0.2177	0.0853	0.1405
3	0.2257	0.0747	0.1237

Figure 19 shows the total power consumption supplied from each microgrid at each time period considering the three microgrids. The graph presents the total power consumption over 24 time periods from each of the three microgrids, averaged across 200 simulated scenarios. Microgrid 2 (red line) exhibits the highest and most variable power output, with pronounced peaks during late afternoon and evening hours. Microgrid 3 (green line) shows similarly fluctuating behavior, though with slightly lower magnitudes. In contrast, Microgrid 1 (blue line) maintains consistently low power contributions across all time periods, indicating a limited generation role or a targeted supply strategy. The average consumption curve (purple line) reflects aggregate demand dynamics, with elevated levels during mid-morning and early evening—periods typically associated with peak residential energy use. These trends align with the previously observed routing behaviors and load factor metrics, confirming that Microgrids 2 and 3 serve as primary distributors, while Microgrid 1 provides stable, low-volume support.

### Impact of microgrid outages on system performance

To evaluate the resilience and the robust performance of the proposed mesh architecture, the system was subjected to N-1 and N-2 contingency analysis. These scenarios simulate the loss of one and two microgrids, respectively, testing the network’s ability to maintain service continuity under significant structural failure. This analysis aims to assess how the system reconfigures its energy distribution strategy in response to reduced supply capacity, and to quantify the impact on key performance metrics such as load factor and hourly consumption profiles. Understanding these dynamics is critical for validating the robustness of the optimization model under failure scenarios and for informing contingency planning in real-world microgrid deployments.

System Resilience is quantified by the network’s ability to redistribute load dynamically to prevent service interruptions during N-1 and N-2 contingency events. In this study, the resilience metric is specifically defined by the feasibility of the stochastic optimization. Since the model successfully converged for all 600 scenarios under contingency events without requiring load shedding, the system demonstrates 100% resilience (uninterrupted service) through dynamic reconfiguration. Conversely, System Stability is measured by the variance of the Load Factor across stochastic realizations, with lower variance indicating a more predictable and reliable state.

The following results compare system behavior across three operational instances—full availability (three microgrids (MG1, MG2, and MG3)), single microgrid outage (two microgrids (MG1 and MG2)), and dual microgrid outage (one microgrid (MG2))—highlighting shifts in load allocation, network topology, and individual microgrid performance.

**Fig. 20:**
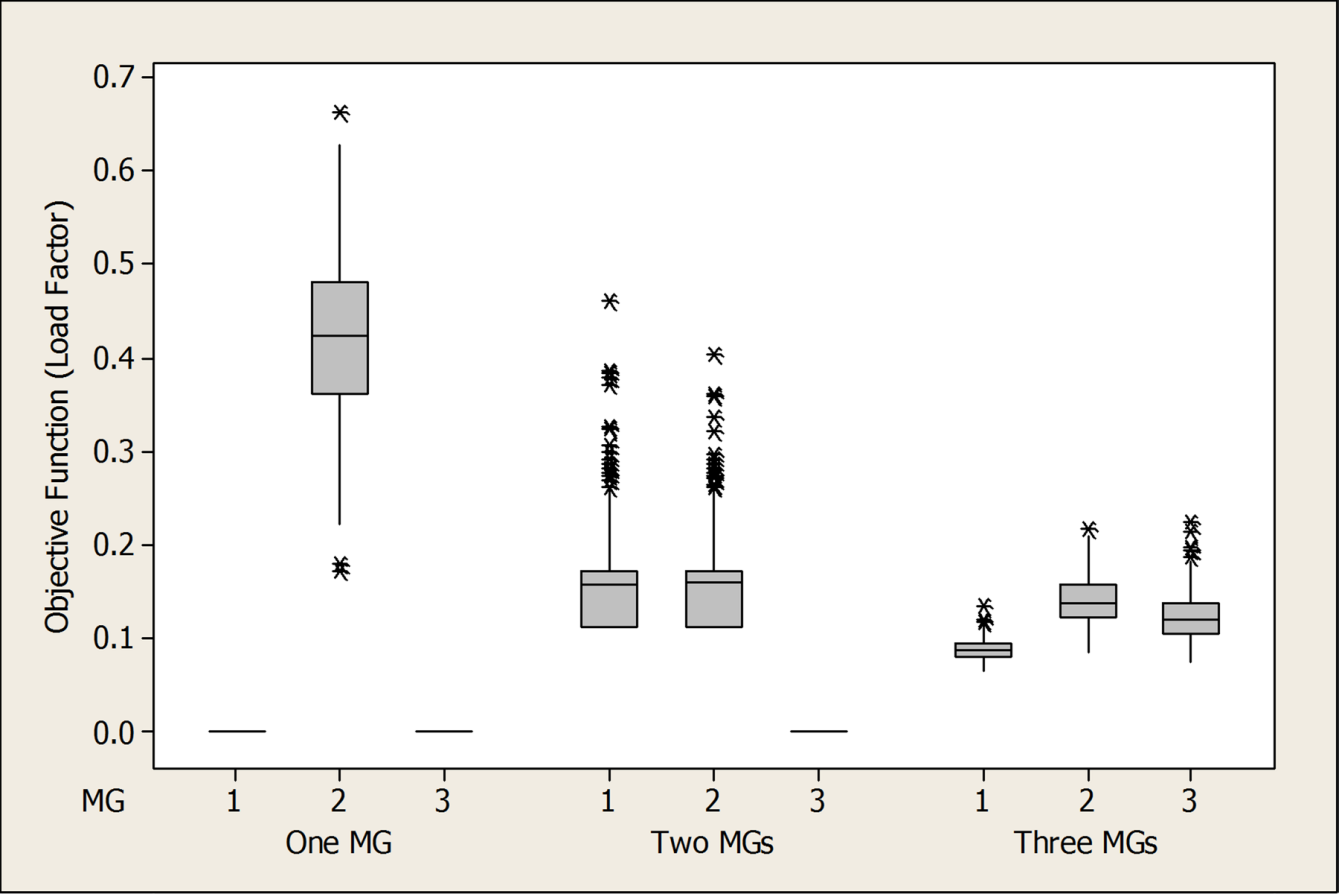
Boxplots of One MG, Two MGs, Three MGs highlight the impact of microgrid activation on Load Factor.

Table 5; Fig. 20 provide a comparative assessment of load factor performance across three operational scenarios: full microgrid availability, single microgrid outage, and dual microgrid outage. As shown in Table 5, the average load factor values increase notably as the number of active microgrids decreases. For instance, under the one-microgrid scenario, MG 2 exhibits an average load factor of 0.4226—nearly triple its value under full availability (0.1405)—indicating a significant increase in operational burden. This trend reflects the system’s compensatory behavior, where remaining microgrids absorb additional load to maintain service continuity.

**Table 5:** Load factor values over the 200 scenarios considering different settings.

	Microgrid	Max load factor value	Min load factor value	Average load factor value	Stability Metric (Variance)
One MG	2	0.6623	0.1731	0.4226	0.0075
Two MGs	1	0.4605	0.1128	0.1597	0.0029
	2	0.4032	0.1128	0.1604	0.0033
Three MGs	1	0.1355	0.0648	0.0880	0.0001
	2	0.2177	0.0853	0.1405	0.0007
	3	0.2257	0.0747	0.1237	0.0007

Figure 20 illustrates the impact of microgrid availability on network performance using box plots of the load factor across three operational settings: one, two, and three active microgrids. The analysis reveals a clear trend: the median load factor is highest under the single-microgrid scenario (0.425), reflecting a concentrated performance where one microgrid bears the full system load. However, this configuration also exhibits the greatest variability, with a wide range of values (0.223–0.627) and elevated variance, indicating less predictable and potentially unstable behavior. Actually, the increased vertical spread (interquartile range) in the single-microgrid scenario represents a quantifiable decrease in operational stability compared to the three-microgrid configuration. In contrast, the three-microgrid setting yields lower median load factors (0.087, 0.138, and 0.12 for MGs 1, 2, and 3, respectively) but demonstrates significantly tighter distributions and reduced volatility. This contrast highlights a fundamental trade-off in network design: while a consolidated system may offer higher peak performance, it does so at the expense of consistency, whereas a decentralized configuration ensures more stable and reliable outcomes across time. While the system achieves full resilience by averting load shedding, the higher variance resulted in ONE MG instance (see, Table 5) indicates a transition toward a more volatile state. This highlights the ‘stress-testing’ capability of the proposed stochastic model and confirms that the integrated mesh topology is essential for maintaining a predictable steady-state under uncertainty.

**Fig. 21:**
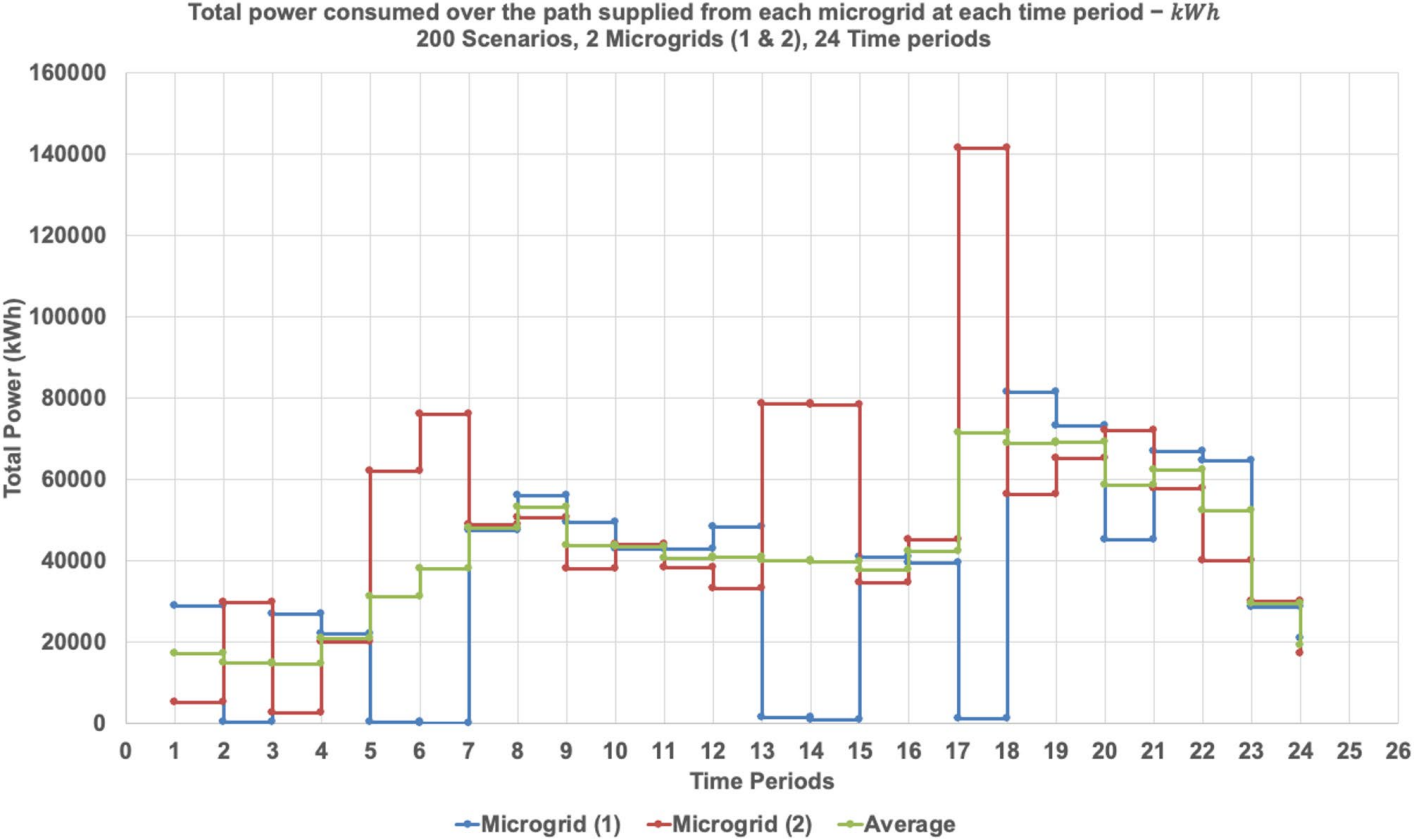
Total power consumed over each path supplied from each microgrid at each time period considering two microgrids (1, and 2) − ℎ.

**Fig. 22:**
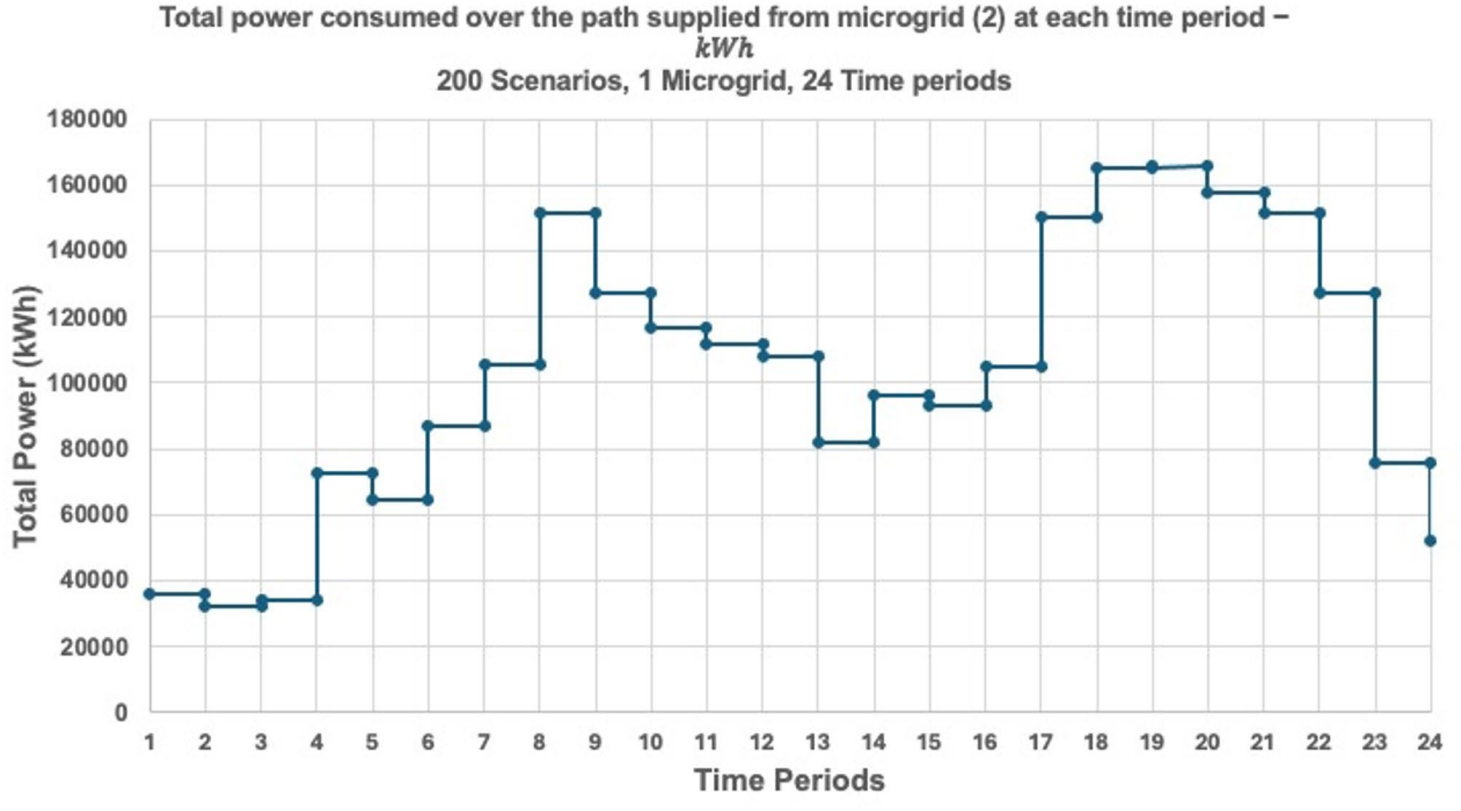
Total power consumed over each path supplied from microgrid (1) at each time period − ℎ.

**Fig. 23:**
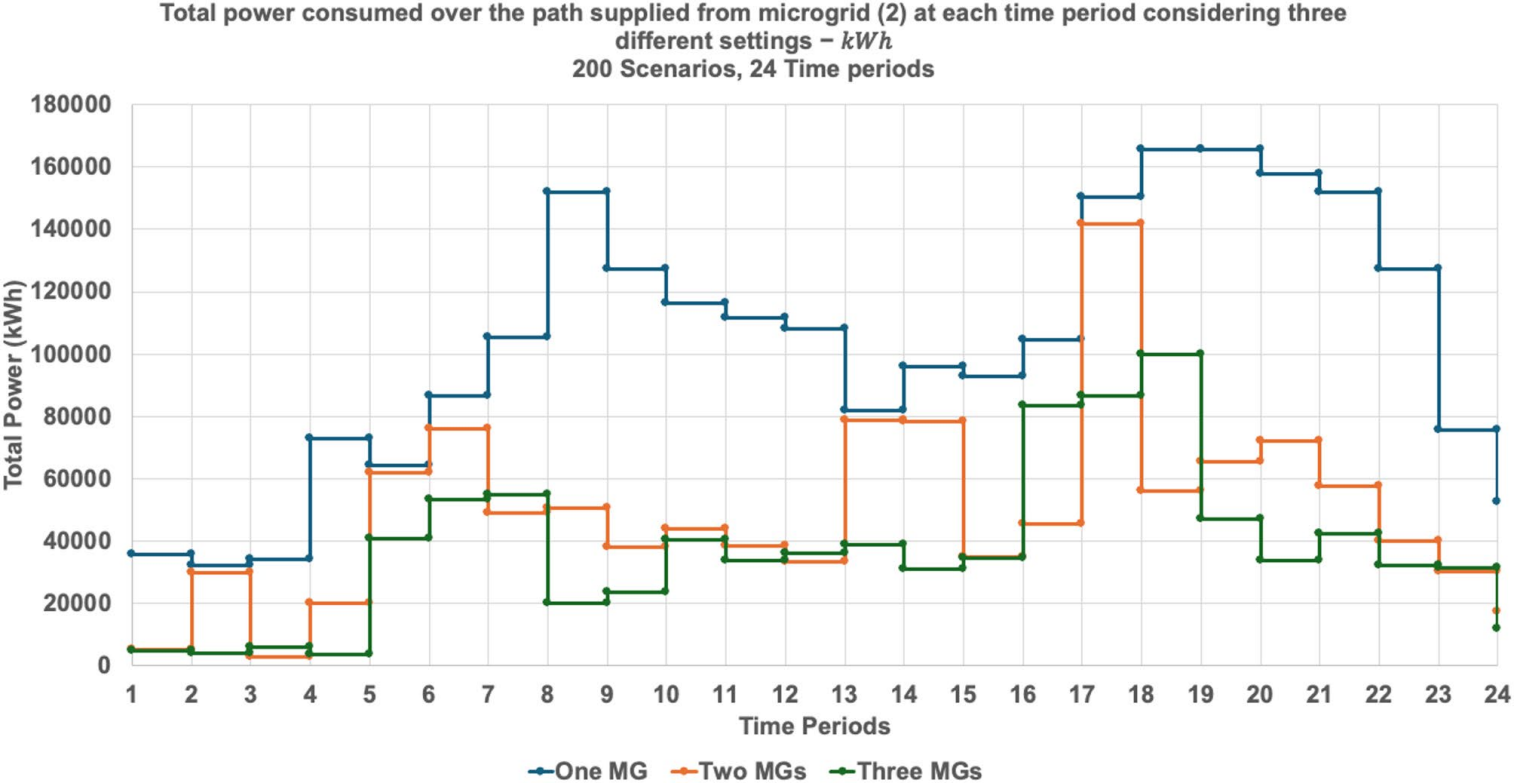
Total power consumed from microgrid (1) at each time period considering the three settings.

Figures 21 and 22, and 23 collectively illustrate how total power consumption evolves across 24 time periods under varying microgrid availability scenarios. Figure 21 shows the load profile when one microgrid is shut down. Microgrid 2 compensates heavily, with sharp peaks reaching over 140,000 kWh around period 16, while Microgrid 1 remains relatively stable. This indicates a significant shift in demand toward MG 2, underscoring its central role in maintaining system continuity during partial outages. Figure 22 presents the single-microgrid scenario, where MG 2 alone supplies the entire network. The load curve exhibits pronounced fluctuations and peak loads exceeding 170,000 kWh, especially during periods 14, 18, and 21. This reflects high operational stress and confirms the volatility observed in the load factor analysis. Figure 23 compares MG 2’s load profile across the three scenarios—full, partial, and minimal availability. The curve steepens significantly as fewer microgrids are active, highlighting MG 2’s increasing burden. This reinforces the insight that while MG 2 can absorb demand, its performance becomes less predictable and more variable under resource constraints.

**Fig. 24:**
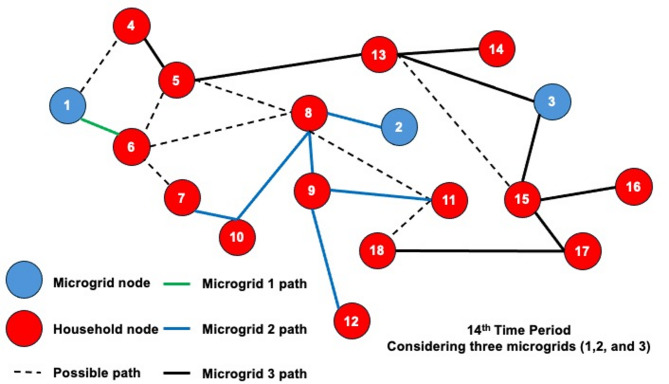
Network configuration for a time period (14th period) considering 200 scenarios and 3 microgrids (1, 2, and 3).

**Fig. 25:**
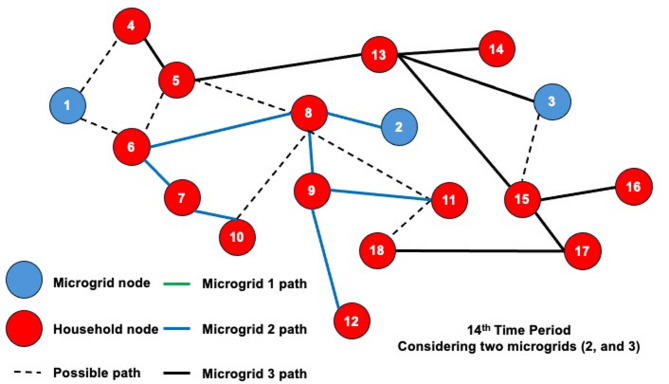
Network configuration for a time period (14th period) considering 200 scenarios and 2 microgrids (1, and 2).

**Fig. 26:**
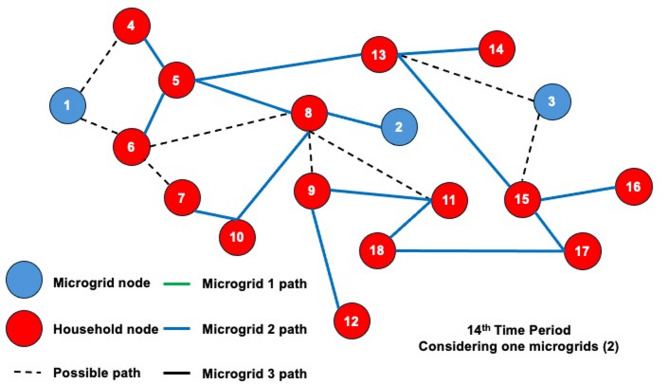
Network configuration for a time period (14th period) considering 200 scenarios and one microgrid (1).

Figures 24 and 25, and 26 illustrate the network configuration at time period 14 across three operational scenarios—three, two, and one active microgrid(s). As microgrid availability declines, the routing structure becomes increasingly centralized, with MG 2 absorbing most of the connections. This shift confirms the system’s adaptive reconfiguration strategy but also reveals reduced path diversity and increased dependence on a single node.

**Fig. 27:**
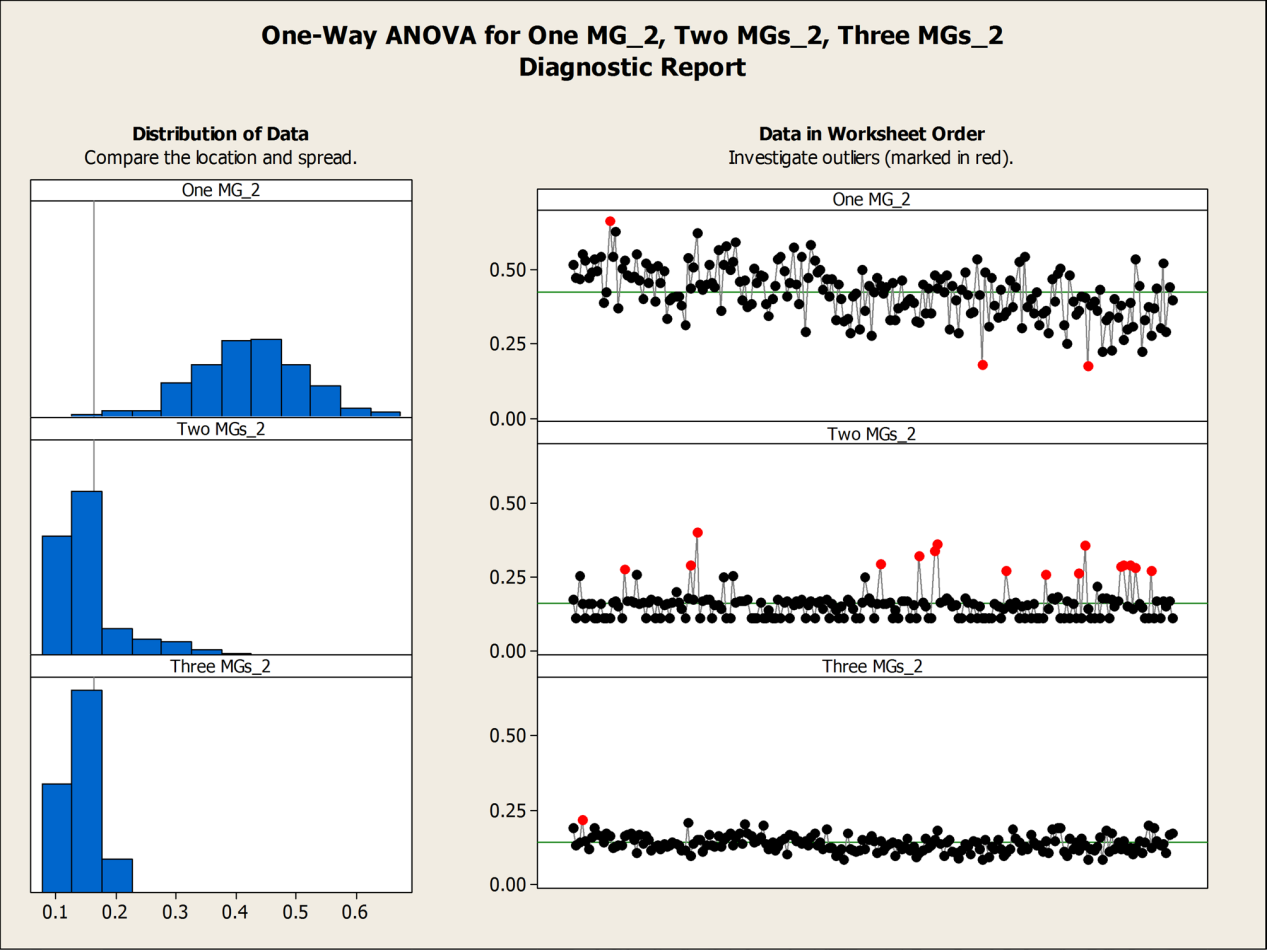
Individual scenario plot (200 Scenario) for MG 2 under Three, Two and One MG instances.

**Fig. 28:**
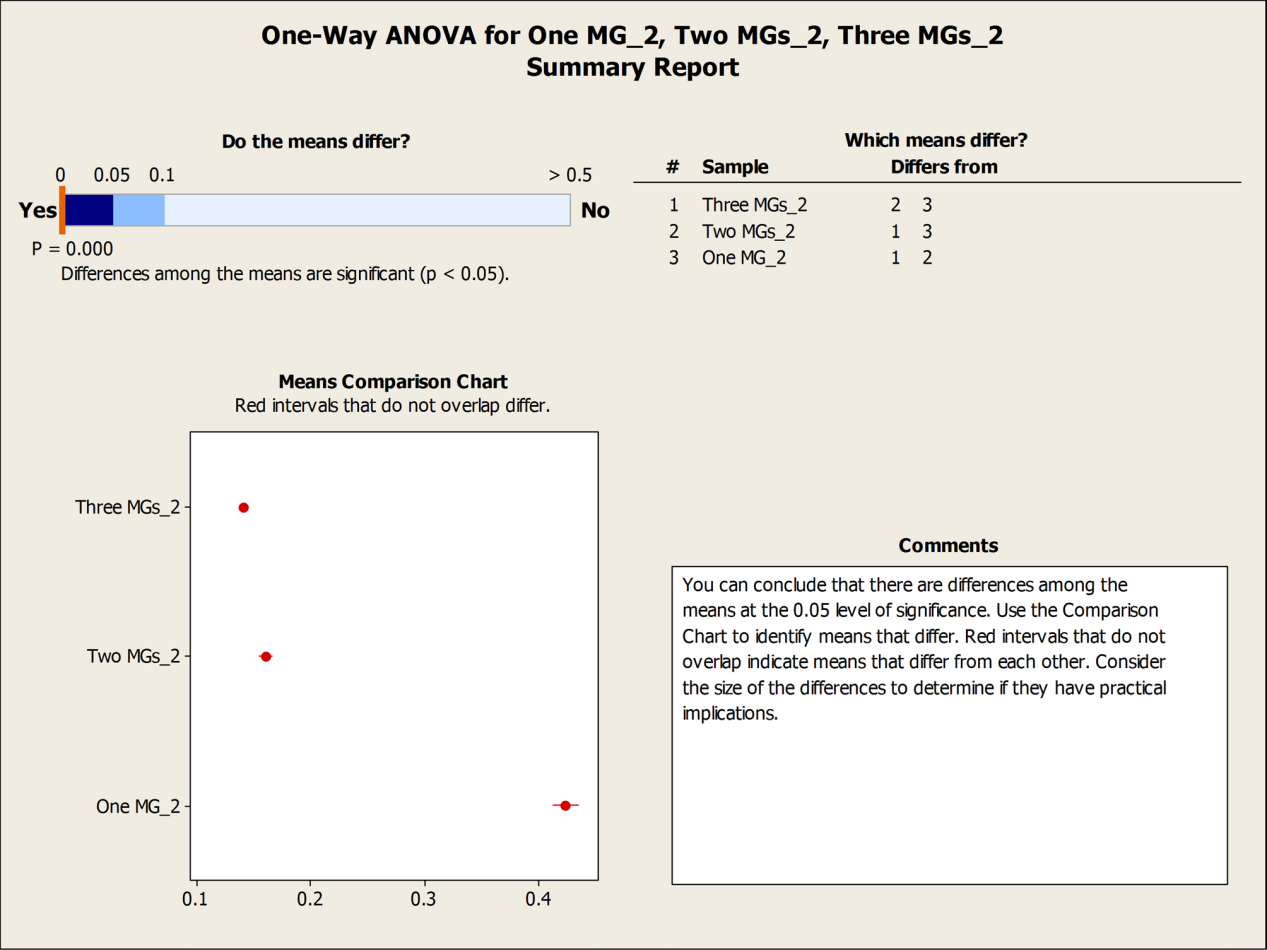
Means Comparison chart for MG 2 under Three, Two and One MG instances.

Figures 27 and 28 collectively highlight the stabilizing effect of microgrid integration on system performance under demand uncertainty. Figure 27 shows the individual load factor values for MG 2 across 200 demand scenarios, revealing substantial dispersion when MG 2 operates alone. This variability underscores its heightened sensitivity to demand fluctuations in the absence of supporting microgrids. Conversely, when MG 2 operates within a fully integrated system (three-MG setting), its load factor values cluster tightly around 0.140, indicating more predictable and balanced performance. Figure 28 reinforces this trend by summarizing the mean load factor values across the three configurations, confirming that increased microgrid availability not only distributes the load more evenly but also enhances operational stability.

Together, these figures highlight a critical trade-off in microgrid design: while the network remains functionally resilient under outage conditions, performance becomes increasingly concentrated and volatile—particularly for MG 2. This contrast underscores a key advantage of interconnected microgrid systems—their ability to absorb and mitigate demand-side disruptions. In a standalone configuration, uncertainty in demand directly amplifies fluctuations in the load factor of the active microgrid, resulting in unpredictable performance. By contrast, integrating multiple microgrids creates a distributed buffer that smooths load variations and promotes stable, predictable operation. This stabilizing effect is statistically validated through analysis of variance (ANOVA), which confirms significant differences among the three configurations (p-value = 0.000), reinforcing the conclusion that system architecture is a critical determinant of operational reliability. The ANOVA test was conducted to evaluate the null hypothesis that microgrid configuration has no impact on system stability. The resulting p-value (p-value ≤ 0.001) allows for the rejection of the null hypothesis, confirming that the mesh-enabled redistribution significantly enhances the stability and reliability of the service.

Ultimately, a fundamental trade-off in network design is revealed: while a consolidated system may offer higher peak performance, it does so at the expense of consistency. Conversely, a decentralized configuration ensures more stable and reliable outcomes across time by distributing the stochastic burden across multiple nodes.

From a financial standpoint, deactivating one or more microgrids may yield immediate cost savings by reducing maintenance, staffing, and fuel costs. The elevated load factor in the single-MG scenario also indicates higher asset utilization, which may appear economically efficient in the short term. However, this efficiency carries increased operational risk. The pronounced variability in load factor under limited configurations signals a heightened likelihood of service disruptions, which could lead to costly instability. Moreover, the absence of redundancy exposes the system to single points of failure, increasing the potential for widespread outages. Thus, while short-term savings may be attractive, they must be carefully weighed against the long-term implications for system resilience, reliability, and risk exposure.

### Managerial implications and methodological limitations

This section provides a critical evaluation of the optimized results, highlights actionable insights for grid management, and identifies the technical constraints of the proposed framework.

The numerical analysis of the microgrid configurations reveals a fundamental stability-efficiency trade-off. It shows that while consolidated single-microgrid operation maximizes the load factor index (increasing asset utilization), this technical efficiency is accompanied by a significant decrease in system stability, as evidenced by high variance in the objective function. Conversely, the three-microgrid configuration provides a distributed buffer that, while yielding a lower average load factor, ensures high operational predictability and reliability under uncertainty.

From a financial perspective, the deactivation of microgrids is shown to yield immediate short-term savings in maintenance, staffing, and fuel costs. However, these savings must be weighed against the increased operational risk associated with the loss of redundancy. The absence of interconnected supply nodes exposes the network to single points of failure, where localized demand volatility can result in service disruptions. Consequently, a transition toward bidirectional mesh topologies is recommended for grid operators. This architectural shift enables the “compensatory behavior” observed in the case study, where microgrids dynamically absorb each other’s loads during outages, thereby maintaining 100% resilience without the need for load shedding.

While the proposed framework demonstrates superior resilience, the following technical limitations are acknowledged to ensure a critical evaluation of the methodology:

First, the optimization is formulated as an MINLP, which is NP-hard. As the number of household nodes or stochastic scenarios (*S*) increases, the computational time required to find a global optimum grows exponentially, which may pose challenges for real-time applications in extremely large-scale distribution networks. To ensure model tractability for this study, a maximum execution limit of one hour was enforced for all solver runs. Consequently, the reported configurations represent high-quality feasible solutions with identified optimality gaps rather than confirmed global optima. This limitation highlights the inherent trade-off between scenario-based accuracy and computational efficiency, suggesting that further algorithmic acceleration or decomposition techniques may be required for real-time application in larger distribution networks.

Second, the effectiveness of the SAA framework is fundamentally contingent upon the availability of a high-volume, high-resolution dataset. As a sampling-based approach, the method seeks to approximate true expectations by averaging over discrete realizations. Sensitivity analysis indicates that a sufficiently large sample size (e.g., *S* = 200) is required to reduce the standard deviation of the objective function and ensure a robust solution. This data-intensive requirement distinguishes SAA from alternative methods such as IGDT, which is non-probabilistic and can function effectively even when historical data is insufficient or probability density functions are unknown. Furthermore, while Robust Optimization typically relies on worst-case bounds and can be less dependent on the total number of scenarios, it often yields overly conservative solutions compared to the balanced, probabilistic insights provided by SAA. Consequently, the proposed model’s performance may be compromised in regions where comprehensive historical load and generation data are unavailable.

Third, households are modeled as intermediate routing nodes, serving as a simplifying abstraction for smart-switch interfaces at the residential level. It is acknowledged that in traditional utility-scale and larger distribution systems, these nodes would typically represent substations or buses rather than individual households acting as pass-through points. However, this approach is used to evaluate the performance of a highly granular mesh architecture in which power flow can be dynamically rerouted at the residential interface to maximize the minimum expected load factor. Implementing such a granular mesh-topology in a real-world setting would necessitate sophisticated smart-switching infrastructure and advanced protection systems. These requirements entail a higher initial capital investment than traditional radial configurations, which may limit the framework’s immediate applicability in cost-sensitive regions. As this study focuses primarily on system-wide management and stochastic optimization, the detailed electrical engineering specifications of these hardware interfaces are currently outside the research scope. It is anticipated that these technical aspects will be addressed in future research through collaboration with electrical engineering specialists to validate the hardware-level feasibility of this granular reconfiguration strategy.

## Conclusions and future research

A stochastic MINLP framework has been proposed to solve the dynamic reconfiguration problem in mesh-connected multi-microgrid networks. The model explicitly accounts for uncertainties in residential demand and renewable generation through an empirical probability distribution derived from two years of historical data. The primary objective is the maximization of the minimal expected load factor across the network, ensuring a robust distribution of operational stress. The SAA method is employed to ensure proactive network management. Through sensitivity analysis, an optimal sample size of 200 scenarios is identified to minimize objective function variance and ensure solution robustness.

The effectiveness of the proposed framework has been validated through N-1 and N-2 contingency analyses. A single-microgrid configuration significantly improves the load factor but introduces high operational volatility and reduced predictability. Conversely, a fully integrated three-microgrid architecture enhances system stability and resilience by acting as a distributed buffer that smooths demand variations, despite yielding a lower load factor. It is demonstrated that the system achieves 100% resilience, as service continuity is maintained for all households without the need for load shedding during microgrid outages. A fundamental stability-efficiency trade-off is revealed: while consolidated configurations maximize asset utilization, decentralized mesh architectures significantly enhance operational stability. This stabilizing effect is statistically confirmed through ANOVA (p-value = 0.000).

From a financial perspective, deactivating microgrids may yield short-term savings in maintenance and fuel costs. However, these benefits are offset by increased operational risks and the loss of redundancy, which are vital for mitigating single points of failure.

While the framework provides a robust solution, several methodological limitations are acknowledged. The optimization problem is characterized by high computational complexity, leading to identified optimality gaps when a one-hour execution limit is enforced. Furthermore, the effectiveness of the SAA method is contingent upon high-resolution datasets; it is observed that a sample size of (*N* = 200) is required to minimize variance in the objective function. Additionally, the modeling of households as intermediate routing nodes serves as a simplifying abstraction for smart-switch interfaces, necessitating higher initial capital investment for hardware and protection systems compared to traditional radial configurations.

Future research will investigate the integration of energy storage systems and dump loads to further mitigate renewable intermittency. Moreover, interdisciplinary collaboration with electrical engineering specialists is anticipated to validate the hardware-level feasibility and protection coordination of granular mesh reconfigurations. This study provides a foundational management framework for achieving resilient, demand-side integrated energy distribution in the next generation of smart neighborhoods.

## Data Availability

The data supporting the findings of this study are included in the manuscript. Additional details are available upon reasonable request from the corresponding authors. For data access, please contact Mohamed Gheith (mohamed.gheith@ejust.edu.eg) (primary contact) or Zakaria Yahia (zakaria.yahia@ejust.edu.eg).

## Abbreviations

MG: Microgrid
SAA: The sample average approximation
KWh: Kilowatt-hours
IEA: The International Energy Agency
DSM: Demand side management
DR: Demand response
DER: Distributed energy resources
IGDT: Information gap decision theory
MGR: Micro grid reconfiguration
MMG: Multi-Microgrid
NR: Network reconfiguration
MMGR: MMG reconfiguration
MINLP: Mixed-integer nonlinear programming
DNR: Distribution network reconfiguration
DG: Distributed generator
HSA: Harmony search algorithm
PABC: Particle artificial bee colony algorithm
IMG: Islanded microgrid
AMOHSA: Adaptive multi-objective harmony search algorithm
PV: Solar photovoltaic
GA: Genetic algorithms
ISSA: Improved slap swarm algorithm
TLBO: Teaching-learning-based optimization algorithm
EPSO: Evolutionary particle swarm optimization
PSO: Particle swarm optimization
MOHBB-BC: Multi-objective hybrid big bang-big crunch
SBF: Self-adaptive bacterial foraging algorithm
ECOST: Expected interruption costs
DA: Dragonfly algorithm
DGUA: Distributed generation units allocation
MILP: Mixed-integer linear programming
LF: Load factor
ANOVA: Analysis of variance

## Sets and indices

$\:h$: $\:\mathrm{Index}\text{}\mathrm{for}\text{}\mathrm{households\:}\{\mathrm{1,2},\dots\:..,H\}$, where *H* is the total number of households.
$\:m$: $\:\mathrm{Index\:for\:microgrids\:}\{\mathrm{1,2},\dots\:,M\}$, where *M* is the total number of microgrids.
$\:i\ and\ j$: $\:\mathrm{Index\:for\:nodes\:in\:the\:network\:}\{\mathrm{1,2},\dots\:,N\}$, where *N* is the total number of nodes including microgrids and microgrids ($\:N=M\cup\:H)$
$\:t$: $\:\mathrm{I}\mathrm{ndex\:for\:time\:slot\:}\{\mathrm{1,2},\dots\:,T\}$, where *T* is the total number of time periods
$\:DC\left(i\right)$: Set of nodes which are on the path that supply node $\:\left(i\right)$
$\:S$: Sample size or the total number of scenarios for stochastic modelling
$$\:s$$: $$\:\mathrm{Index\:for\:uncertainty\:scenarios}\:\left\{\mathrm{1,2},\dots\:,S\right\}.$$

## Symbols

$\:{D}_{hts}$: Demand (i.e. energy consumed) of household $\:h$ at time *t* under scenario *s* (kWh)
$\:{C}_{mts}$: Capacity (i.e. total amount of energy produced) of Microgrid $\:m$ at time *t* under scenario *s* (kWh)
$\:{L}_{ijm}$: Resistance of the branch $\:(i-j)$ supplied from microgrid $\:\left(m\right)-{\Omega\:}/m$
$\:{Tloss}_{s}$: The total power loss over the whole network considering scenario $\:\left(s\right)\:-\:kW$
$\:{\widehat{I}}_{its}$: The current needed for household $\:h$ at time *t* under scenario *s* (Amp)
$\:{x}_{ijmt}$: A binary decision variable which is equal to 1 if node $\:\left(i\right)$ is connected to node $\:\left(j\right)$ supplied from microgrid $\:\left(m\right)$ at time period $\:\left(t\right)$, and 0 otherwise
$\:{y}_{imt}$: A binary decision variable which is equal to 1 if node $\:\left(i\right)$ is supplied form microgrid $\:\left(m\right)$ at time period $\:\left(t\right)$, and 0 otherwise
$\:{I}_{imts}$: The total current over the path from microgrid $\:\left(m\right)$ to node $\:\left(i\right)$ at time period $\:\left(t\right)$ considering scenario $\:\left(s\right)-Amp$
$\:{loss}_{mts}$: The energy loss over the path supplied from microgrid $\:\left(m\right)$ at time period $\:\left(t\right)$ considering scenario $\:\left(s\right)-kWh$
$\:{W}_{mts}$: Total energy consumed over the path supplied from microgrid $\:\left(m\right)$ at time period $\:\left(t\right)$ considering scenario $\:\left(s\right)-kWh$
$\:{E}_{ms}$: Total energy consumed over the paths supplied from microgrid $\:\left(m\right)$ over the 24 h considering scenario $\:\left(s\right)-kWh$
$\:{AVG}_{ms}$: Average energy consumed over the path supplied from microgrid $\:\left(m\right)$ considering scenario $\:\left(s\right)-kWh$
$\:{MaxD}_{ms}$: Maximum power demand for microgrid $\:\left(m\right)$ considering scenario $\:\left(s\right)-kW$
$\:{LF}_{ms}$: Load factor for microgrid $\:\left(m\right)$ considering scenario $\:\left(s\right)$
